# Genome-wide nucleotide patterns and potential mechanisms of genome divergence following domestication in maize and soybean

**DOI:** 10.1186/s13059-019-1683-6

**Published:** 2019-04-25

**Authors:** Jinyu Wang, Xianran Li, Kyung Do Kim, Michael J. Scanlon, Scott A. Jackson, Nathan M. Springer, Jianming Yu

**Affiliations:** 1 Department of Agronomy, Iowa State University, Ames, IA 50011 USA; 2 Center for Applied Genetic Technologies, University of Georgia, Athens, GA 30602 USA; 3 Plant Biology Section, School of Integrative Plant Science, Cornell University, Ithaca, NY 14853 USA; 4 Department of Plant and Microbial Biology, University of Minnesota, St. Paul, MN 55108 USA

**Keywords:** Evolution, Domestication, Base composition, Genome divergence, Solar UV, Mutation, Methylation, UV damage repair

## Abstract

**Background:**

Plant domestication provides a unique model to study genome evolution. Many studies have been conducted to examine genes, genetic diversity, genome structure, and epigenome changes associated with domestication. Interestingly, domesticated accessions have significantly higher [A] and [T] values across genome-wide polymorphic sites than accessions sampled from the corresponding progenitor species. However, the relative contributions of different genomic regions to this genome divergence pattern and underlying mechanisms have not been well characterized.

**Results:**

Here, we investigate the genome-wide base-composition patterns by analyzing millions of SNPs segregating among 100 accessions from a teosinte-maize comparison set and among 302 accessions from a wild-domesticated soybean comparison set. We show that non-genic part of the genome has a greater contribution than genic SNPs to the [AT]-increase observed between wild and domesticated accessions in maize and soybean. The separation between wild and domesticated accessions in [AT] values is significantly enlarged in non-genic and pericentromeric regions. Motif frequency and sequence context analyses show the motifs (PyCG) related to solar-UV signature are enriched in these regions, particularly when they are methylated. Additional analysis using population-private SNPs also implicates the role of these motifs in relatively recent mutations. With base-composition across polymorphic sites as a genome phenotype, genome scans identify a set of putative candidate genes involved in UV damage repair pathways.

**Conclusions:**

The [AT]-increase is more pronounced in genomic regions that are non-genic, pericentromeric, transposable elements; methylated; and with low recombination. Our findings establish important links among UV radiation, mutation, DNA repair, methylation, and genome evolution.

**Electronic supplementary material:**

The online version of this article (10.1186/s13059-019-1683-6) contains supplementary material, which is available to authorized users.

## Background

Domestication is a special mode of evolution. Extensive studies have been carried out to understand the domestication process and genes associated with morphological changes [1–4]. Meanwhile, genomes also went through profound changes during domestication. Recent studies documented the base-composition difference and mutation rate difference between populations separated by either domestication or demographic bottleneck event, which provide novel insights on genome evolution [5–7]. Further investigation in DNA base composition, mutation spectrum, and the potential relationship between them is necessary to advance our understanding of genome changes.

DNA base composition is an essential genomic feature. Remarkable research progress has been made in several areas, including codon usage bias [8], isochore structure [9, 10], and GC-biased gene conversion [11]. Recently, a conserved base-composition pattern, modern accessions having significantly higher [A] and [T] values across genome-wide polymorphic sites than accessions sampled from their wild relatives, was discovered with natural populations across multiple species [5]. Different genomic regions exhibit different patterns of a number of genomic features such as DNA methylation, GC content, and recombination rate [12–15]. It would be interesting to study the regional variation of genome change pattern, captured by base composition summarized from polymorphic sites.

Mutation is a fundamental factor that generates the genetic variation upon which selection, drift, and recombination act. Point mutations are the most common type of mutations with a universal bias toward high AT, primarily due to the high rate of transition mutations [16]. Recent studies indicated that mutation rate can be different across populations [6, 7]. Divergence in mutation rates or types between populations are one of several factors that affect genetic variation patterns [17]. Analysis of data from multiple mutation accumulation experiments, either accumulating spontaneous or induced mutations, demonstrated higher [AT] values across mutation sites in derived lines at the end of mutation experiments than in ancestral lines, which suggested that base-composition difference can emerge from mutation sites [5]. Characterization of mutation spectrum in natural populations may help unravel the mechanism of genome change [18].

Organisms have evolved a complex system to monitor and repair DNA damage caused by various exogenous mutagens, such as solar-ultraviolet (UV) radiation, reactive oxygen species, excess boron or aluminum, and pathogenic microorganisms [19]. For plants, solar-UV radiation is a major exogenous mutagen as they use sunlight for photosynthesis. The primary solar UV-induced DNA lesion, cyclobutane pyrimidine dimers (CPDs), induces C→T base transitions [20]. CPDs distort the DNA’s double-helix structure, which influences DNA unwinding and DNA replication, and ultimately affect cell cycle [21]. Using sets of SNPs private to different human populations, a recent study suggested that UV might have been involved in the mutation spectrum change [6].

DNA methylation is a major form of epigenetic modification in many eukaryotic genomes. It not only regulates gene expression and silences transposons and repeat sequences, but also affects mutation rates [22–25]. DNA methylation occurs in CG, CHG (where H = A, C, or T), and CHH sequence contexts in plants [26, 27]. The relative frequency of DNA methylation varies substantially along chromosome. DNA methylation is primarily distributed in the heterochromatin regions that are mostly composed of tandem repeats and transposons [12, 13, 28]. It has been shown that methylation of cytosine residue at CpG sites can enhance the solar UV-promoted CPD formation [25]. We can ask whether the rate of solar UV-induced mutations varies along the chromosome and whether base composition can summarize such variation.

In this study, we report findings from the analysis of millions of SNPs segregating among 100 accessions from a teosinte-maize comparison set and among 302 accessions from a wild-domesticated soybean comparison set. First, we show that higher [AT] values in domesticated accessions relative to wild accessions, or [AT]-increase, are consistently observed for SNPs found in either genic or non-genic portions of the genome, with non-genic SNPs having a greater contribution to the [AT]-increase. Interestingly, we also find that the divergence in [AT] is much higher in pericentromeric regions than in other regions. All 4 sequence motifs related to solar-UV signature consistently have higher frequencies in methylated regions than unmethylated regions. With a different set of population-private SNPs, we also discover the enrichment of mutations related to the solar-UV signature in domesticated accessions. Using base-composition across polymorphic sites as the phenotype, genome-wide scans identify a set of putative candidate genes involved in UV damage repair pathways. Together, these findings seem to suggest that solar-UV radiation and differential mutation repair are critical components in the genome divergence process that resulted in domesticated accessions’ greater numbers of nucleotides A and T.

## Results

### Genome-wide [AT]-increase

**Fig. 1:**
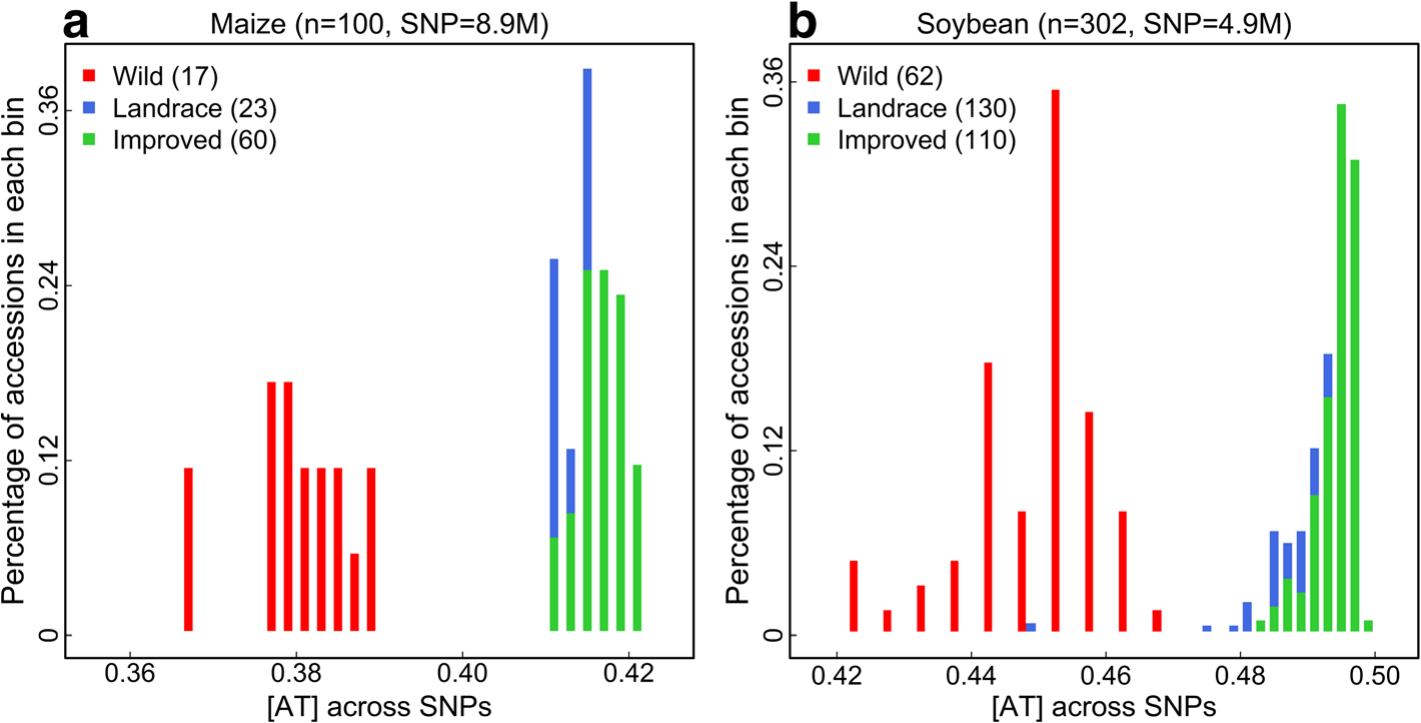
Genome-wide base-composition pattern in maize and soybean. **a** The distribution of [AT] among 8.9 million SNPs in 100 maize accessions. **b** The distribution of [AT] across 4.9 million SNPs in 302 soybean accessions

We obtained a set of 8,852,678 SNPs in 100 teosinte-maize accessions and a set of 4,870,265 SNPs in 302 wild-domesticated soybean accessions from the original studies [29, 30] (Additional file 1: Figure S1). These SNPs are designated as common SNP sets to compute the genome-wide base-composition across polymorphic sites without concerning about sampling issues due to low minor allele frequency (MAF) or high missing rate [5]. For each accession, we obtained an [AT] value calculated as the fraction of SNP alleles that are either base A or T. The choice of [AT] was based on the finding that single-strand parity rule 2 (PR2) applies to base composition across SNPs [5], i.e., [A] ≈ [T] and [G] ≈ [C]. In both maize and soybean sets, wild and domesticated (including landraces and improved cultivars) accessions are clearly separated by [AT] (*P* value is 1.49e^−14^ for maize and 1.02e^−44^ for soybean). Domesticated accessions have more nucleotides A and T at the polymorphic sites (Fig. 1), termed as [AT]-increase (domesticated > wild accessions). In maize, the average value of [AT] in wild accessions is 0.380 (SD = 0.006), while the average values of [AT] in landraces and improved cultivars are 0.414 (SD = 0.003) and 0.417 (SD = 0.003), respectively. In soybean, the average value of [AT] in wild accessions is 0.449 (SD = 0.010), while the average values of [AT] in landraces and improved cultivars are 0.492 (SD = 0.006) and 0.494 (SD = 0.003), respectively.

### Base composition among DNA substitution types

Bi-allelic SNPs can be grouped into six substitution types without defining the ancestral allele. To further understand the consistent [AT]-increase pattern, we examined the contribution to [AT]-increase from each substitution type (Additional file 1: Figure S2). Two transition types, A/G and C/T, are the major types detected in maize and soybean genomes, with each having a frequency of ~ 34%, much higher than the expected frequency by chance (i.e., ~ 17% or 1/6). The average frequency for each of four transversion types (A/C, A/T, C/G, and G/T) is less than 10%, with C/G type being the least frequent one.

**Fig. 2:**
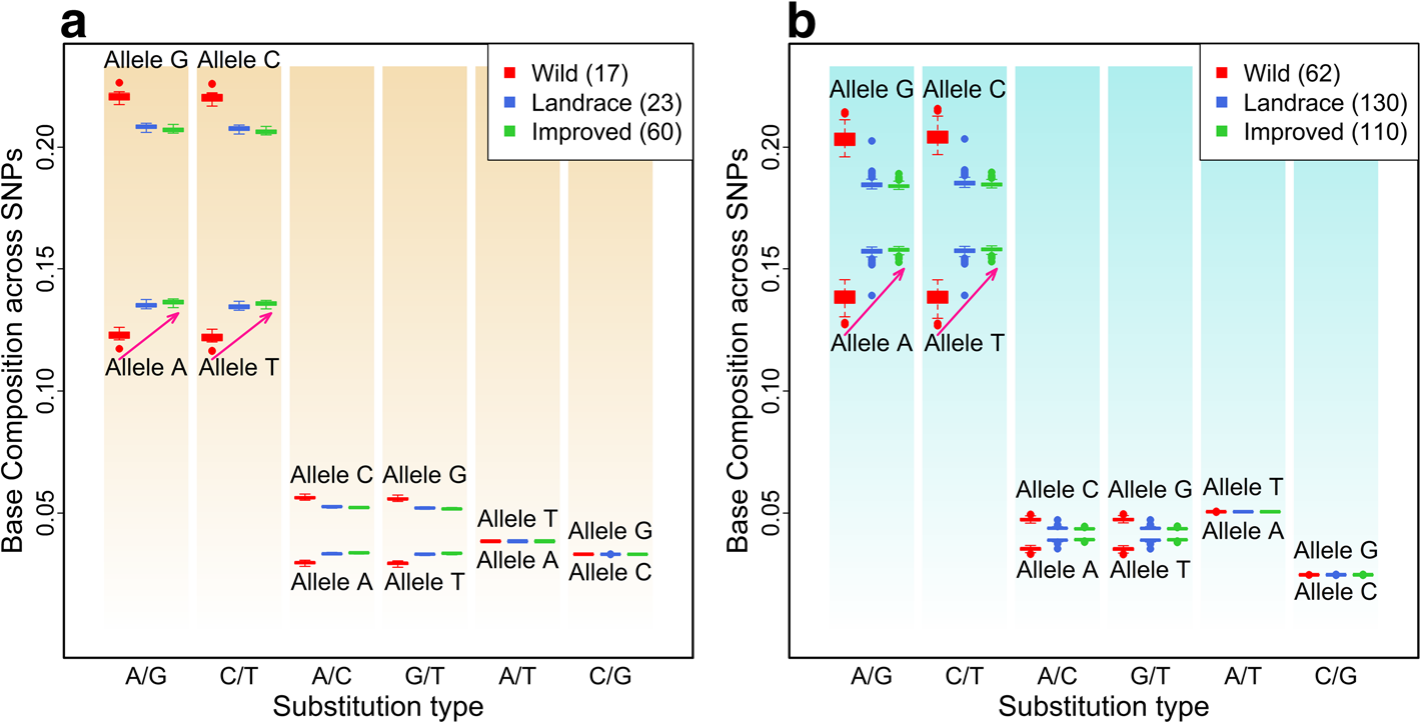
Base-composition distribution at each of the six substitution types in maize (**a**) and soybean (**b**). The genome-wide SNPs were classified into six substitution types. Base composition was calculated for each accession conditional on each substitution type. The red arrows show the [A] and [T] increase at A/G and C/T substitution types

We then calculated the base-composition value across polymorphic sites conditional on each substitution type. The contribution to the overall [AT]-increase varied among substitution types (Fig. 2). Two transition types (A/G and C/T) are the major contributors due to their high frequencies and that the majority of wild accessions possess G or C allele for these types, while the domesticated typically have A or T. For A/C and G/T types, significant base-composition differences between wild and domesticated groups are also evident, and the proportional increase in A or T is similar to that of A/G and C/T types. However, because of their relatively low frequencies (≤9%), these two types contribute less to the overall [AT]-increase. Neither A/T nor C/G type contributes to the overall [AT]-increase.

### Base-composition pattern at different genomic regions

**Fig. 3:**
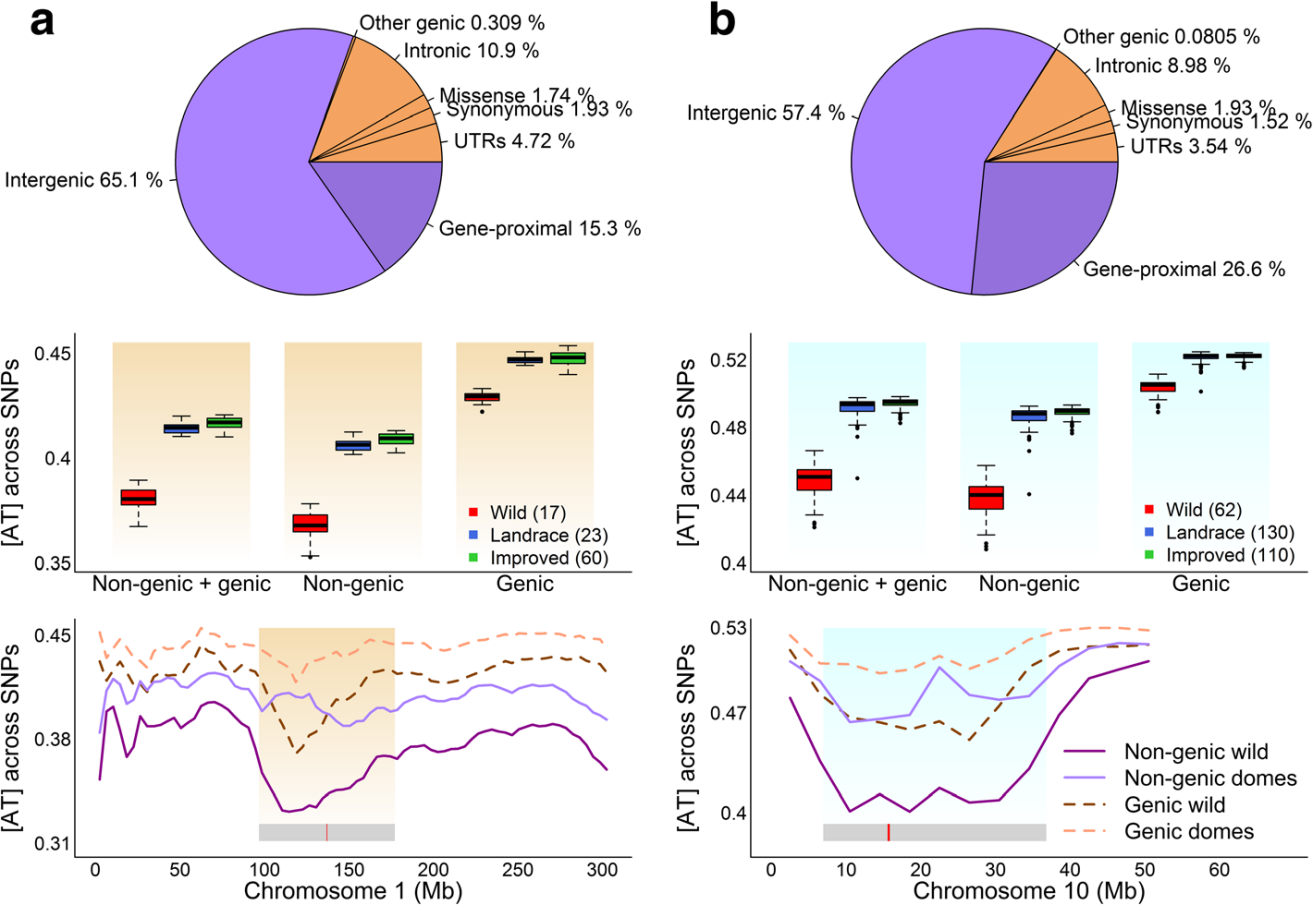
The distribution of base composition calculated with genic and non-genic SNPs in maize (**a**) and soybean (**b**). The upper panel shows the distribution of SNPs across different genomic annotation sets. The middle panel shows the base-composition distribution with genic and non-genic SNPs. The lower panel illustrates the base-composition distribution across 5 Mb segments with genic and non-genic SNPs. To simplify the plot in the lower panel, landraces and improved cultivars are combined to be the domesticated group to compare with the wild group. For each accession, base composition was calculated using a moving average approach with a 5-Mb window size and a 4-Mb step size. Each point in the plot represents the mean [AT] of the specified group across a 5-Mb window. The gray bar in the bottom indicates the position of the pericentromeric region, and the red bar within the gray bar shows the position of the centromeric region

It is known that different genomic regions exhibit different patterns for a number of genomic features including DNA methylation, GC content, and recombination rate [12–15], which naturally led us to investigate the base-composition distribution at different parts of the genome. To facilitate this, we first classified the genome-wide SNPs to 7 genomic annotation sets: intergenic, gene-proximal, UTRs, synonymous, missense, intronic, and other genic [31, 32] (Fig. 3). Intergenic SNPs are the most common group (65.1% in maize and 57.4% in soybean), followed by gene-proximal (15.3% in maize and 26.6% in soybean) and intronic (10.9% in maize and 8.98% in soybean). Because the numbers of SNPs were relatively too small in several genomic annotation sets, we combined intergenic and gene-proximal sets to form the non-genic SNP set and combined the rest five original genomic annotation sets to form the genic SNP set. The non-genic set contains 7,120,981 SNPs in maize and 4,088,443 SNPs in soybean, and the genic set contains 1,731,687 SNPs in maize and 781,822 SNPs in soybean.

We calculated the [AT] value for each accession from genic and non-genic SNP sets separately. [AT] of domesticated accessions is consistently higher than that of wild accessions in both genic and non-genic SNPs (Fig. 3). However, non-genic SNPs have greater contributions to the [AT]-increase, and the [AT]-difference between wild and domesticated accessions is about twice that of genic SNPs. Since the total number of non-genic SNPs are 4 to 5.5 times larger than genic SNPs, we randomly sampled an equal number of SNPs from genic and non-genic SNP sets to obtain the [AT] value for comparison. We obtained a consistent trend from 100 subsets, demonstrating that the greater contribution to the overall [AT]-increase from non-genic SNPs is not only because of its larger SNP number but also due to its higher proportional increase in [AT] than genic SNPs (Additional file 1: Figure S3). As expected, further comparisons of [AT] distribution between missense, synonymous, and intergenic SNP sets (Additional file 1: Figure S4A-B) show that while [AT]-difference between wild and domesticated accessions from missense and synonymous SNP sets are similar to each other, both of them are smaller than intergenic SNP set. We also evaluated the impact of allele frequency on the different contributions from genic and non-genic SNPs. Compared with non-genic SNP set, genic SNP set generally has more SNPs with high MAF and fewer SNPs with low MAF (Additional file 1: Figure S5), which may suggest that the genic region is more conserved than the non-genic regions.

Both species are known to have low gene density in pericentromeric regions [29, 33, 34], so we examined the [AT] distribution with genic and non-genic SNPs along chromosomes (Fig. 3, Additional file 1: Figures S6-S8). Along each chromosome, (a) higher [AT] in domesticated group than wild group is consistently observed for both genic and non-genic SNPs; (b) [AT]-difference between domesticated and wild group for non-genic SNPs is generally larger than that for genic SNPs; and (c) [AT] for each accession is higher for genic SNPs than for non-genic SNPs. More interestingly, the divergence in [AT] is significantly enlarged in the pericentromeric regions, especially for non-genic SNPs.

Because of the dramatic difference of [AT] distributions between pericentromeric regions and chromosome arms, we further compared the [AT] distribution between genic and non-genic regions conditional on the pericentromeric regions and chromosome arms separately (Additional file 1: Figure S4C-F). The [AT]-difference between wild and domesticated accessions at the non-genic region is consistently about twice that of the genic regions for both pericentromeric regions and chromosomal arms. And the [AT]-difference between wild and domesticated accessions at the pericentromeric region is much larger than that of chromosome arms, which is true for both non-genic and genic SNPs.

We speculate the enlarged [AT]-difference in the pericentromeric regions is associated with the fact that these regions mainly consist of repetitive sequences and transposable elements [33–36] that are mostly arranged in heterochromatin [37] and generally have low recombination rates [30, 33, 34, 38]. To verify the speculation, we first examined the distribution of base composition at transposable element (TE) and non-transposable element (non-TE) regions. The [AT]-differences at TE regions are much larger than non-TE regions (Additional file 1: Figure S9). We then plotted the [AT]-difference and crossover rate for maize and recombination rate for soybean along each chromosome (Additional file 1: Figures S10-S12). Negative correlations between [AT]-difference and crossover/recombination rate are significant for all 10 maize chromosomes and 18 soybean chromosomes. We observed relatively low and fluctuating MAF within the pericentromeric regions (Additional file 1: Figures S13-S15), which may be related to the low efficiencies in purging out deleterious alleles [39].

As the phenotypic differences between the wild and domesticated accessions mainly shaped by the artificial selection, we then compared the base-composition distribution at domestication selective sweep and non-selective sweep regions to test if the domestication process was partially responsible for the detected base-composition difference. The [AT]-difference between wild and domesticated accessions at selective sweep regions is much larger than that at non-selective sweep regions (Additional file 1: Figure S16). This suggests that the domestication process indeed have an effect on the detected base-composition difference at the polymorphic sites.

### Enrichment of motifs related to solar-UV signature surrounding SNP sites

**Fig. 4:**
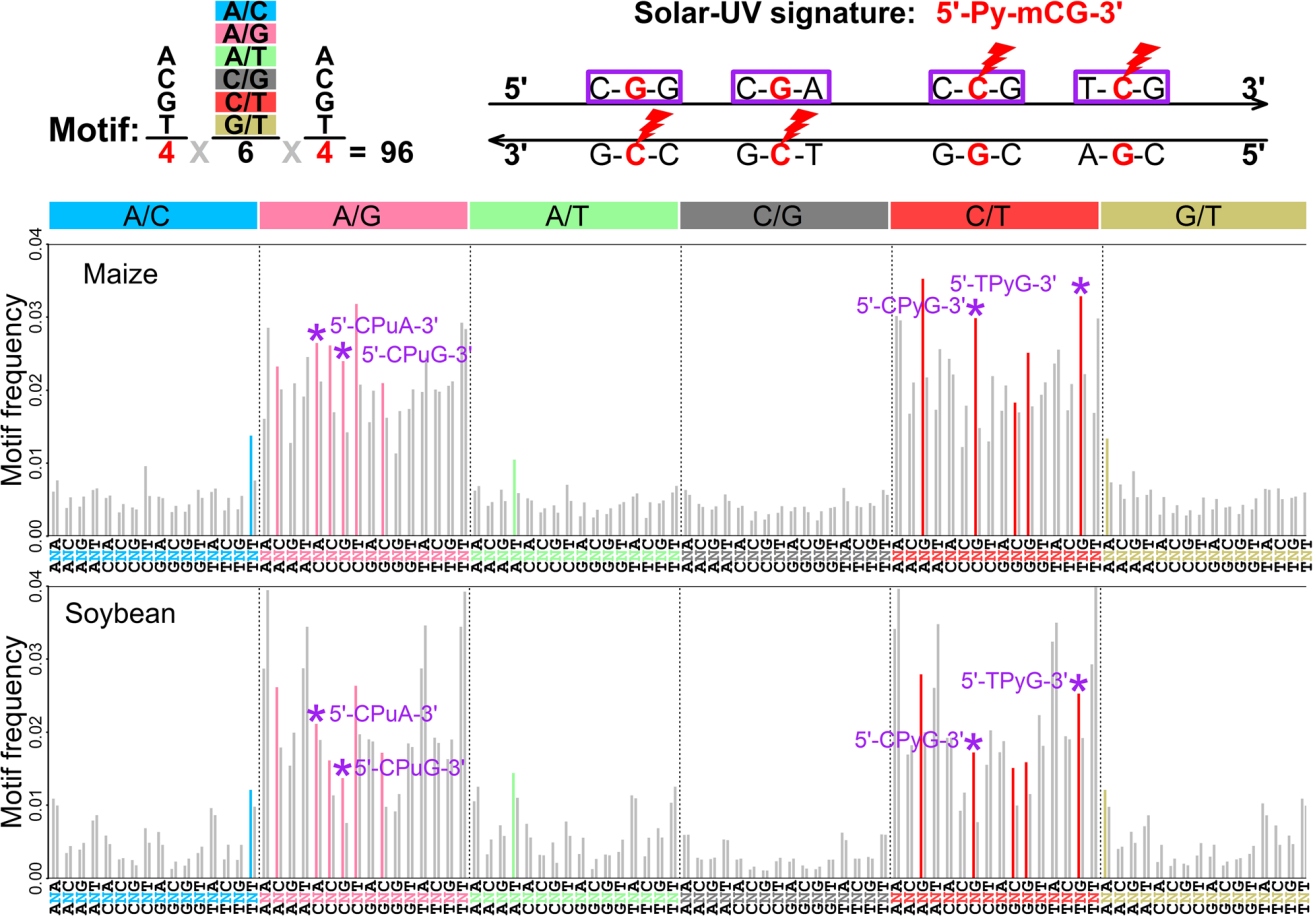
Motif enrichment analysis in maize and soybean. The upper panel illustrates the composition of tri-nucleotide motifs and the induction of motifs related to solar-UV signature on double strand DNA. Each tri-nucleotide motif is formed by incorporating reference base pairs immediately upstream and downstream to the middle SNP site. Ninety-six motifs are divided into 6 classes based on the substitution types of the SNP. The lightning sign shows the mutation site, and the purple rectangle highlights the motifs related to the solar-UV signature. The middle and lower panels show the frequency of motif in maize and soybean, respectively. For each motif, the left bar is the overall frequency around SNP sites, while the right bar is the overall frequency of the same motif around random sites (an empirical 95th percentile drawn from 100 random sample scenarios). The colored bar indicates the common motif between maize and soybean with a frequency greater than 1/96, and the frequency of motif at SNP sites is higher than that at random sites. The bar with a star on top highlights the motif related to solar-UV signature

To test whether SNPs occurred more frequently in certain sequence contexts, we first classified SNPs into 96 tri-nucleotide motifs by considering 1 base directly adjacent upstream and downstream of the SNP site. Then, we examined the frequency and the enrichment of tri-nucleotide motifs. With 96 possible motifs, the expected frequency is 0.010 (≈ 1/96) and a ratio of 1.000 between the frequency of motif at SNP sites and that at random sites if SNPs occurred randomly in the genome. We detected 14 common motifs between maize and soybean with both frequencies and ratios greater than the expected, and 11 out of 14 were from A/G and C/T transition types (Fig. 4). In both species, 5′-CNG-3′ (N is the polymorphic site) around C/T type has the highest ratio with 2.007 in maize and 2.228 in soybean. In addition, 5′-TNG-3′ is enriched around C/T type, with a ratio of 1.477 in maize and 1.311 in soybean. Because most wild accessions have C allele at C/T type (Fig. 2), these SNPs were more likely changed from 5′-PyCG-3′ to 5′-PyTG-3′, where Py is either pyrimidine C or T. Correspondingly, the reverse and complementary motifs 5′-CNG-3′ and 5′-CNA-3′ around A/G type are also overrepresented, which suggests the high chance of 5′-CGPu-3′ to 5′-CAPu-3′ mutations, where Pu is purine G or A.

Solar UV induces CPDs preferentially at 5-methylcytosine-containing dipyrimidine sites (5′-Py-_m_CG-3′), termed as solar-UV signature [20, 40]. Thus, the overrepresented motif 5′-PyCG-3′ around C/T (the reverse and complementary motif 5′-CGPu-3′ around A/G) is the same as the solar-UV signature if C is methylated. Hereafter, we refer to the four aforementioned sequence motifs as motifs related to solar-UV signature. In both species, _m_CG level is negatively correlated with gene density and enriched in the pericentromeric regions [12, 13, 28, 41], which suggests that motifs related to solar-UV signature might occur more frequently outside of the genic regions and be overrepresented in the pericentromeric regions. To test this, we performed two sets of comparisons: frequencies of motifs related to solar-UV signature between genic and non-genic SNPs, and between SNPs from pericentromeric and non-pericentromeric regions. As expected, all four motifs related to solar-UV signature have higher frequencies within non-genic SNP sets than genic SNP sets, and they have higher frequencies among SNPs from pericentromeric regions than among SNPs from non-pericentromeric regions (Additional file 1: Figures S17-S18).

We then examined the role of DNA methylation by calculating the frequencies of motifs related to solar-UV signature conditional on methylated and unmethylated regions [42–44]. We found that all four motifs related to solar-UV signature consistently have higher frequencies in methylated regions than in unmethylated regions with genic SNPs, non-genic SNPs, SNPs from pericentromeric regions, and SNPs from non-pericentromeric regions (Additional file 1: Figures S17-S18). This suggests the higher probability of C→T and G→A transitions, potentially stimulated by DNA methylation, in non-genic regions and pericentromeric regions, which agrees with our findings of non-genic SNPs’ larger contributions to [AT]-difference and the enlarged [AT]-difference in pericentromeric regions.

### Mutation spectra of population-private variation

The findings of sequence motifs related to solar-UV signature enriched in common SNP sets encourage us to verify the pattern with rare segregating SNPs that occurred as relatively recent mutations [45, 46]. Therefore, following the procedures laid out in a previous study [6], we compiled private SNP sets that contain 2,651,790 population-private SNPs in maize and 681,791 population-private SNPs in soybean from original studies [29, 30] (Additional file 1: Figure S1). These private SNP sets are different from the earlier common SNP sets with a small overlap. A SNP is considered as population private if it is segregating in 1 lineage but fixed ancestral allele in other lineages. For each crop, we obtained 4 population-private SNP sets: private wild SNPs (PW), private domesticated SNPs (PD), private landrace SNPs (PL), and private improved cultivar SNPs (PI). PW designates SNPs that are segregating in the wild group but are fixed ancestral alleles in the landrace and the improved cultivar groups; PL means those SNPs are segregating in the landrace group but are fixed ancestral alleles in the wild and the improved cultivar groups, and similarly for other private SNP sets. Analyzing such SNPs enables us to assess the mutation rate difference among different lineages after diverged from the most recent common ancestor.

**Fig. 5:**
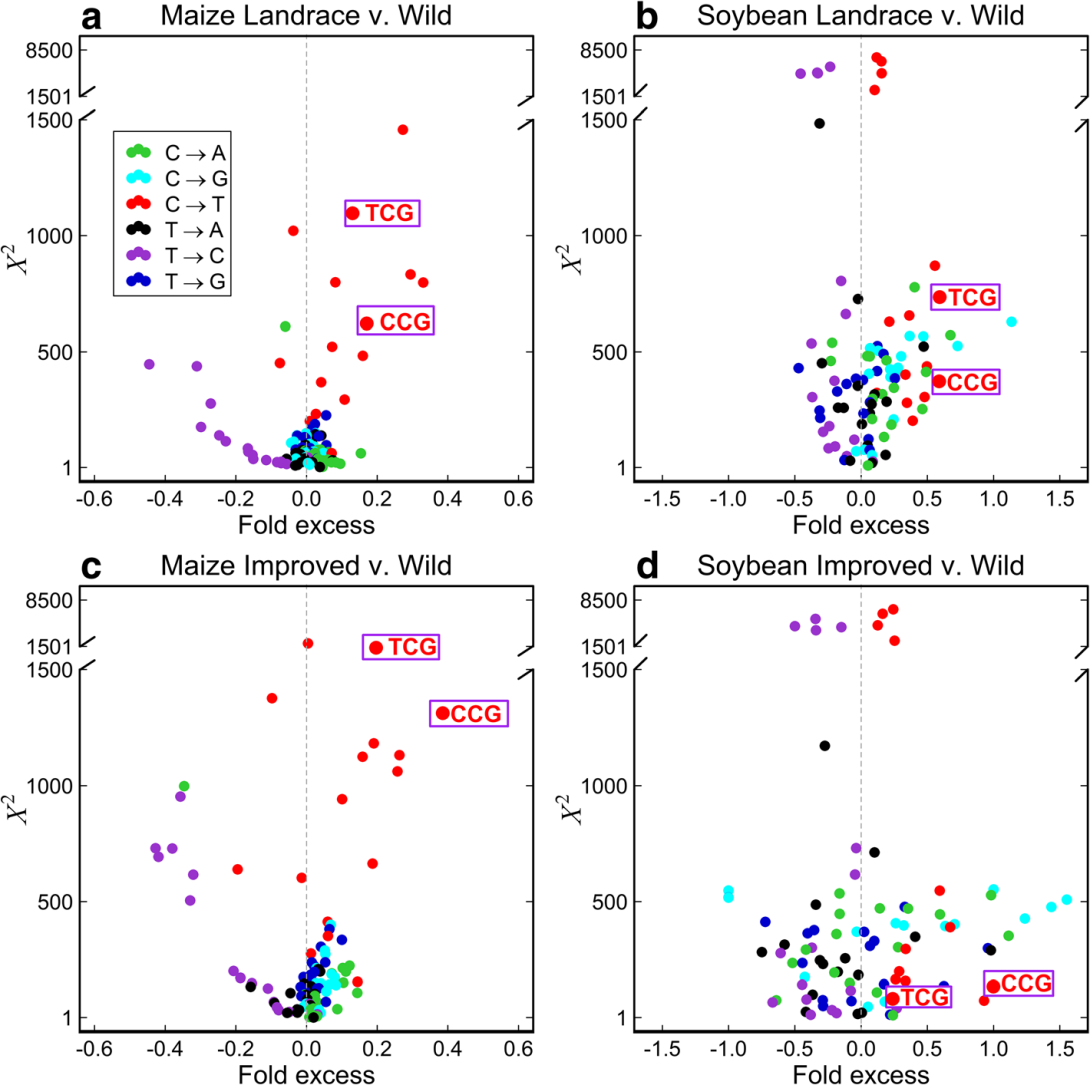
Enrichment test of mutations related to solar-UV signature with population-private SNPs. **a**, **b** Compare the mutation frequency between landraces and wild accessions in maize and soybean, respectively, and the *x* coordinate of each point indicates the fold frequency difference (*f*_PL_(*m*) − *f*_PW_(*m*))/*f*_PW_(*m*). **c**, **d** Compare the mutation frequency between improved cultivars and wild accessions in maize and soybean, respectively, and the *x* coordinate of each point indicates the fold frequency difference (*f*_PI_(*m*) − *f*_PW_(*m*))/*f*_PW_(*m*). The *y* coordinate indicates Pearson’s *χ*^2^ value that measures the significance of the difference between *f*_*m*_(*P*_1_) and *f*_*m*_(*P*_2_). Outlier points are labeled with the ancestral state of the mutant nucleotide flanked by two neighboring bases, and the color of the points indicate the ancestral and derived alleles of the mutant site. The purple rectangle highlights the mutations related to solar-UV signature. Here, TCG on the plot represents mutation 5′-TCG-3′→5′-TTG-3′ and its reverse complement 5′-CGA-3′→5′-CAA-3′, CCG represents mutation 5′-CCG-3′→5′-CTG-3′ and its reverse complement 5′-CGG-3′→5′-CAG-3′, and similarly for all the other dots on the plot

Next, we tested the differences in the spectrum of mutagenesis between populations with population-private variants as described in the previous study [6]. With ancestral allele information, population-private SNPs can be partitioned into 96 mutation types by considering the base immediately upstream and downstream of the variable site [47]. In both species, most C→T transitions have higher frequencies in PL and PI than in PW, which agrees with the previous finding in a human study [6] (Fig. 5). This observation suggests although genomes of domesticated and wild accessions were continuing to evolve after divergence, domesticated accessions might have higher C→T mutation rate. We observed a higher rate of mutations related to solar-UV signature 5′-TCG-3′→5′-TTG-3′ and 5′-CCG-3′→5′-CTG-3′ (hereafter abbreviated as TCG→T and CCG→T) in domesticated accessions than in wild accessions (Fig. 5, Additional file 1: Figure S19). For instance, in maize, TCG→T has a frequency of 3.45% in PL and 3.55% in PI compared with 2.99% in PW. The higher frequencies of TCG→T and CCG→T in domesticated than wild accessions are consistent for all chromosomes (Additional file 1: Figure S20). We further split each population-private SNP set to genic-private SNPs and non-genic-private SNPs, and pericentromeric-private SNPs and non-pericentromeric-private SNPs. As shown by Additional file 1: Figure S21, in both species, the TCG→T and CCG→T mutations generally have higher frequencies with non-genic-private SNPs and pericentromeric-private SNPs.

This overrepresentation of mutations related to solar-UV signature found in the private SNP sets together with the enrichment of motifs related to solar-UV signature found in the common SNP sets suggests that solar UV is potentially one of the major forces driving the [AT]-increase pattern during domestication.

### Overrepresentation of genes repairing UV-damaged DNA near loci associated with genome divergence

With genome-wide association studies (GWAS), the previous study in human found the enrichment of DNA repair genes surrounding loci associated with genome divergence captured by base-composition across polymorphic sites [5]. The enrichment of solar-UV signature mutations in domesticated accessions suggests that solar-UV radiation plays an important role in driving the [AT]-increase pattern. Plant genomes encode a complex system to monitor and repair DNA damage. We assessed whether genes involved in UV damage repair pathways are enriched near loci associated with genome divergence for [AT].

Using the [AT] values obtained from the common SNP sets as a genome phenotype, GWAS identified a series of loci significantly associated with base-composition across polymorphic sites (Additional file 1: Figure S22). Based on either the sequence similarity of rice genes or *Arabidopsis* genes [48], 334 maize and 107 soybean genes were compiled as related to UV-damaged DNA repair (UV-related gene hereafter). Proportion tests indicate that the UV-related genes were more likely to reside nearby GWAS signals than by chance (Additional file 1: Tables S1-S4). In maize, for the 500-kb segments around significantly associated SNPs, we identified 4.2% of UV-related genes, but these regions only encode 1.8% of all annotated genes. In soybean, for the 500-kb segments around significantly associated SNPs, 20.6% of UV-related genes were identified, while only 13.8% of annotated genes were encoded in these regions. The tagged genes involved in all the steps for global genome nucleotide excision repair (NER) pathway to repair UV damage are shown in Additional file 1: Figure S23.

**Fig. 6:**
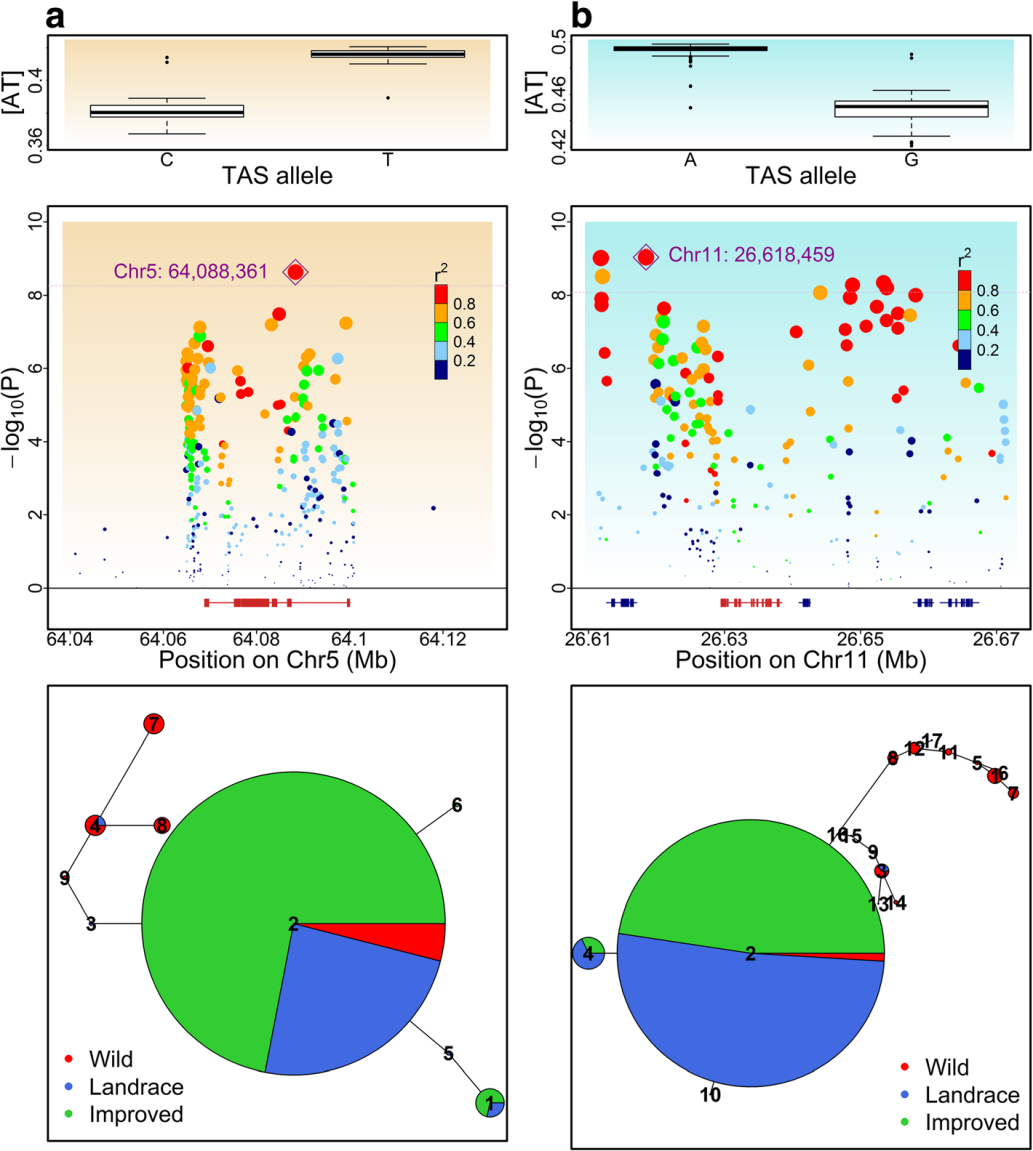
UV-related DNA repair genes implicated by trait-associated SNPs (TASs) and haplotype demographic distributions. **a** *ATR* in maize is tagged by a TAS (PZE0561610418) on chromosome 5. **b** *DNA ligase1* (*Lig1*) in soybean is tagged by a TAS (rs1126618459) on chromosome 11. The upper panel shows the box plot of base composition between accessions carrying different alleles at the TASs. The middle panel shows the regional Manhattan plot around *ATR* and *Lig1* locus (*ATR* and *Lig1* are shown in red, others in blue). Dot size is proportional to the magnitude of significance for the SNP’s association with [AT] variation. Dot color indicates its LD with the TAS. The lower panel shows the haplotype networks inferred from 8 SNPs within *ATR* gene and 16 SNPs within *Lig1* gene, respectively. Each circle represents one haplotype. Size of the circle is proportional to the number of accessions possessed the haplotype. Size of each colored slice within a circle is proportional to the number of accessions possessed the haplotype from the corresponding group

We performed a detailed analysis of several UV-related genes located near significant GWAS SNPs (Fig. 6). A SNP located within maize *ATR* (*Zm00001d014813*) is significantly associated with base-composition across polymorphic sites. The *ATR* encodes a putative ATR protein which functions in a wide range of responses to DNA damage, including sensing and activating a cell cycle arrest in response to UV-B-caused DNA damage [19]. We found eight nonsynonymous variants located in *ATR* in this maize population*.* In soybean, a SNP located 11 kb downstream of *Ligase1* (*Glyma.11g193100*, *Lig1*) on chromosome 11 is strongly associated with [AT] variation. *Lig1* in soybean encodes a putative DNA ligase 1 protein which functions in sealing the nick of DNA at the last step of the repairing process. Besides one nonsense and two nonsynonymous SNPs, we also detected a 1.8-kb deletion at the fifth intron in wild soybean accessions (Additional file 1: Figure S24). Soybean genome encodes two copies of *Lig1*, and we did not detect signals for *Lig1* on chromosome 12.

Both *ATR* and *Lig1* are located within selective sweep regions identified in previous studies [30, 49], which suggest the possibility that polymorphisms within *ATR* and *Lig1* went through domestication bottleneck. We then conducted haplotype network analysis of these two genes. There are two distinct clusters of haplotypes in both *ATR* and *Lig1* (Fig. 6), one composed mostly of domesticated accession haplotypes and the other composed mostly of wild accession haplotypes. We refer to these clusters as domesticated cluster haplotype (DCH) and the wild cluster haplotype. In *ATR*, DCH is present in > 98% of maize but < 18% of teosinte; while in *Lig1*, DCH is present in > 97% of domesticated soybean but < 5% of wild soybean. Intriguingly, the major haplotype (haplotype2) in both genes are shared by most of the domesticated accessions and a small number of wild accessions. Haplotype2 in *ATR* is shared among 86.7% of maize and 17.6% of teosinte, and haplotype2 in *Lig1* is shared by 86.7% of domesticated soybean and 2% of wild soybean. Considering that domestication largely involved selection of favorable alleles from standing allelic variation in wild ancestors [1], it is likely that the major haplotypes for both *ATR* and *Lig1* were present in the ancestral populations with low frequency, and their frequencies increased rapidly during domestication.

## Discussion

Our understanding of how plant genomes have changed following domestication bottlenecks remains limited. In this study, we aim to address the question from a novel angle by surveying the genome-wide base-composition pattern and its potential associated mechanisms. Focusing on a genome phenotype summarized from millions of polymorphic sites along the chromosome, we provide novel insights on genome evolution at different parts of the genome: genic versus non-genic, pericentromeric versus non-pericentromeric, and methylated versus unmethylated. This study also presents a first case where a few critical components in genome evolution are brought together: “base composition”, “mutation”, “UV radiation”, “DNA repair”, and “methylation”.

The [AT]-increase in domesticated over wild accessions is consistently observed with the overall genome-wide SNPs, SNPs within major genomic annotation sets, and SNPs from different genomic regions. These findings indicate the presence of common underlying mechanisms that drive the domesticated accessions to build their genomes with more A and T nucleotides.

Demographical analyses have shown that plant and animal species experienced population size changes associated with domestication and range expansion [50–54]. The effective population size of maize has decreased strikingly from the onset of domestication (≈ 10,000 years ago) to the recent past (≈ 1100–2400 years ago) and increased during post-domestication expansion [50]. In contrast to maize, the wild *parviglumis* experienced an increase in effective population size which also lasts until the recent past (≈ 1100–1800 years ago) [50]. In plants, the increased mutational load has been observed in populations that undergo declines in effective population size [50, 55, 56]. Thus, one interpretation for our findings is that domesticated populations have historically lower effective population size, which results in a stronger genetic drift, and consequently lead to higher mutation numbers compared with their wild relatives. Meanwhile, our discovery of the overrepresentation of mutations related to a solar-UV signature in domesticated accessions indicated a varied mutation rate across populations. Therefore, an alternative interpretation is that alleles of UV damage repair genes have different repair efficiency (lower in domesticated accessions) and affect the number of de novo mutations in different lineages.

Regarding the increased [AT] in domesticated accessions, one natural question to ask is: What is the consequence of building genomes with more A and T nucleotides? One possibility will be more efficient energy usage. Energy usage efficiency is a trait under universal selection that has shaped various genomic aspects. For example, highly expressed proteins use cheaper amino acids [57–60] and are generally shorter than lowly expressed ones [61, 62]. Synthesizing a G+C basepair requires a larger amount of energy and nitrogen than producing an A+T basepair [63]. Base stacking for G and C is more energetically expensive compared with that for A and T, as G binds to C with three hydrogen bonds while A binds to T with two hydrogen bonds [64]. Therefore, it may be interesting to ask whether domesticated accessions build their genomes with more A and T so that more energy is saved for other biological processes toward better yield potential.

Recent studies have shown the high heterogeneity of mutation rate across genomic regions [65–67]. Our survey discovered the enrichment of motifs related to solar-UV signature surrounding SNPs, especially for SNPs located in non-genic and pericentromeric regions, which suggests solar-UV radiation is likely one of the major contributors for plant genome divergence. In general, DNA methylation level of non-genic regions is higher than that of genic regions, and pericentromeric regions higher than non-pericentromeric regions [13, 28]. Higher methylation levels in non-genic and pericentromeric regions potentially provide a greater amount of base materials for solar UV-induced C→T transition at the 5′-Py-_m_CG-3′ context, which is also supported by our findings of higher frequencies of motifs related to the solar-UV signature from methylated regions than unmethylated regions. DNA methylation is highly enriched within transposable elements and repetitive sequences [12, 13, 28]. Thus, this interesting connection between DNA methylation and solar UV-induced mutation propels us to ask a critical question: Is the frequent transition of methylated C to T actually a cost that genomes have to pay for having transposons and repetitive sequences methylated?

Compared with chromosome arms, pericentromeric regions are highly enriched with repetitive sequences and transposable elements and generally have higher methylation levels, lower gene density, and lower recombination rates [13, 28, 30, 33–36]. In this study, we observed associations between [AT]-difference and methylation level, transposable element, and recombination rate. A previous study illustrated that DNA transposon activity is associated with an increased number of mutations in the sequences close to the transposon [68]. This suggests that enriched transposable elements at pericentromeric regions may contribute to the increased accumulation of mutations within these regions. In sexual organisms, non-recombining regions of a genome were found to be subjected to Muller’s ratchet [69–72], and regions with active recombination are more efficient in the purging of the deleterious mutations [39]. This may also partially explain the findings of enriched mutations related to solar-UV signature and enlarged [AT]-difference in the pericentromeric regions.

Solar UV primarily induces C→T base transition at 5′-Py_m_CG-3′ sequence context [20, 40, 73], and CG methylation can enhance solar-UV-induced mutation at 5′-Py_m_CG-3′ sites [25]. However, a few questions still need to be addressed to understand the increased rate of mutations related to a solar-UV signature in domesticated accessions. The first question is how DNA methylation varies across populations as variation in DNA methylation level may lead to the observed difference in the rate of mutations related to solar-UV signature between domesticated and wild groups. A recent study on 51 diverse maize inbred lines identified 172 maize-teosinte differentially methylated regions (DMRs), which are biased toward more examples of higher methylation levels in teosinte than maize [74]. Because those DMRs only represent a very small portion of the genome and the majority of the methylated regions are conserved within the maize, the identified DMRs should not be a major contributor to the observed difference in the rate of mutations related to solar-UV signature between the two groups. The other question is how UV could induce germline mutations as germline cells are generally shielded from direct solar radiation. The damaging effects of solar UV are often limited to the epidermis cells due to low UV-B penetration into plant tissues through flavonoid layer [75, 76]. However, some evidences suggest that UV-B may penetrate into meristematic tissues as increased genome instability in plant germline has been observed even with low UV-B radiation [77]. In addition, plant germline cells divide several times during the vegetative growth stage and separated into sex-specific lineages only during late flower development [78]. Thus, we suspect that mutations induced by solar UV during vegetative growth in cells of the apical meristem may be inherited into the progeny.

Using a phenotype summarized from millions of SNPs, we identified a set of UV-related genes nearby signals associated with genome divergence. We speculate at some point before domestication, during gametogenesis, spontaneous mutations randomly took place within a UV-related gene. The gene with altered sequence may have a mild difference in terms of locating or repairing DNA errors [79]. Therefore, the lineages in which mutations in UV-related genes were segregating began to accumulate systematic difference in DNA repair, which contributed to the genome divergence patterns captured by base composition. In the mutation accumulation experiments, once an *Escherichia coli* lineage acquired 1 bp insertion in *mutT* gene at the 26,500th generation, the later generations from this lineage began to show greatly elevated mutation rates and bias toward substitution type from A to C than the progenies from other lineages [73]. The recent study that compared the accumulated mutations after 20 generations between wild-type and DNA repair-deficient mice suggested different patterns in rate and direction between 2 lineages [80]. A similar phenomenon has been observed for somatic mutations in cancer cell. The substitution type and rate vary for patients with different variations in DNA repair genes [81]. The varied mutation rate has been reported in natural populations at the genome level [82], the family level [83], and the subpopulation level [6]. These findings suggested the hypothesis that polymorphisms within UV-related genes played a role in different DNA repair efficiency, which in turn affected the mutation rate differently in different lineages.

Initiation of domestication typically involved a set of key genes controlling for domestication syndrome, a set of traits differentiating wild and domesticated accessions. The causal polymorphisms underlying the domestication syndrome are sought to be the direct targets of artificial selection [1, 3, 4]. Although the UV-related genes were detected through a genome phenotype clearly separated between domesticated and wild accessions, we speculate that these genes were probably not the direct targets because these polymorphisms were less likely to lead to visible agronomic traits that human ancestors desired. The observation that wild and domesticated accessions share the same haplotype for *ATR* and *Lig1* suggested that the polymorphisms in these two genes more likely emerge earlier than the onset of domestication. The consequence of changing these UV-related genes probably promoted the occurrence of desired traits, which was subject to the direct selection. The identified UV-related genes indicate almost every step in the NER pathway contributes to the overall [AT]-increase (Additional file 1: Figure S23), suggesting the complexity of molecular mechanisms.

Molecular experiments need to be carried out to provide evidence supporting the function of these UV-related genes and their connection to the base-composition pattern. Although it is beyond the scope of this study to address the functional difference between wild and domesticated alleles and the molecular mechanisms affecting the repair efficiency, this study pointed to a new direction for addressing some fundamental questions about the genome itself. We think that mutation repair genes, like *ATR* and *Lig1*, harboring significant changes such as altered gene structure, should be the next priority to study and provide molecular evidences. Induced mutation accumulation experiments with UV as the mutagen and near-isogenic lines (NILs) segregating only at the regions surrounding mutation repair genes as starting materials will be preferable to demonstrate the connection between UV-induced mutation and base composition change. Sequencing lines that derived from starting materials carrying mutations at UV-damaged DNA repair gene regions may also provide additional support.

## Conclusions

Base-composition difference between domesticated accessions and wild accessions at the dynamic part of the genome suggests the important role of AT-bias mutation in shaping the overall pattern of base-composition variation. Regional variations of base-composition pattern indicate that non-genic SNPs and pericencentromeric regions have greater contributions to the observed pattern. This finding together with the discovery of solar UV’s potential role in driving the genome divergence establishes the connection between DNA methylation and base-composition variation. By focusing on the evolutionary outcome, our genome scans in maize and soybean identified a set of UV damage repair genes. Rapidly improved genomics and epigenomics capacity would further facilitate our efforts to probe potential connections among base composition, mutation, methylation, DNA repair, and genome evolution.

## Methods

### Sequence information and SNP extraction

In maize, the original SNP set with B73 genome (AGPv2) as references was obtained from 103 maize genomes of Maize Hapmap2 (19 wild accessions, 23 landraces, and 61 improved cultivars) [29]. Three lines, 2 wild accessions and 1 improved cultivar, were removed due to low sequence coverage and a small number of SNPs. In soybean, the original SNP set with Williams 82 genome (version 1.1) as references was obtained from 302 soybean genomes (62 wild accessions, 130 landraces, and 110 improved cultivars) [30]. Information for maize and soybean accessions are provided in Additional file 1: Table S5 and Table S6, respectively. With CrossMap v0.2.5 [84], genome coordinates of the original SNP sets in B73 AGPv2 and Williams 82 version 1.1 were converted to that in B73 AGPv4 and Williams 82 version 2.0, respectively. In maize, the assembly chain file for CrossMap is available at ftp://ftp.ensemblgenomes.org/pub/plants/release-39/assembly_chain/zea_mays/AGPv2_to_AGPv4.chain.gz. And in soybean, the assembly chain file is available at ftp://ftp.ensemblgenomes.org/pub/plants/release-39/assembly_chain/glycine_max/V1.0_to_Glycine_max_v2.0.chain.gz.

Then, for each species, we obtained 2 sets of SNPs (common SNP set and population-private SNP set) from the original SNP sets by applying different filtering criteria (Additional file 1: Figure S1). The common SNP sets containing 8,852,678 SNPs in maize and 4,870,265 in soybean are obtained by filtering with a MAF threshold of 5% and a missing rate threshold of 20%. These common SNP sets are used for all analyses except population-private SNP analysis.

For population-private SNP sets, we followed the procedure laid out in a previous study [6] to obtain 2,651,790 population-private SNPs in maize and 681,791 population-private SNPs in soybean. The private SNP sets are different from the common SNP sets with a small overlap. Ancestral state of the maize allele was inferred based on the allele of *Tripsacum* [49]. To infer the ancestral state of the soybean allele, BLASTN [85] (version 2.2.28+) was used to identify the orthologous regions between soybean and *Medicago truncatula*. Each SNP and its 58 bases flanking sequences were extracted from the soybean genome then blasted to the *Medicago truncatula* genome sequence [86] with an *e* value <1e^−1^ and only the best hit was considered. A SNP is considered as population private if it is segregating in 1 group but fixed ancestral allele in other groups. Based on this definition, we obtained 1,137,732 private wild SNPs (PW) that are segregating in the wild group but fixed ancestral allele in the landrace and improved cultivar groups; 1,514,058 private domesticated SNPs (PD) that are segregating in either the landrace or improved group but fixed ancestral allele in the wild group; 270,390 private landrace SNPs (PL) that are segregating in the landrace group but fixed ancestral allele in the wild and improved cultivar groups; and 537,259 private improved cultivar SNPs (PI) that are segregating in the improved cultivar group but fixed ancestral allele in the wild and landrace groups. In soybean, we obtained 571,756 PW, 110,035 PD, 20,543 PL, and 1798 PI. The total numbers of SNPs (2,651,790 in maize and 681,791 in soybean) in private SNP sets are obtained by summing up PW and PD because there are no overlapping SNPs between the two population-private SNP sets by definition.

For maize, all analyses were done using maize B73 genome (version AGPv4) as references. For soybean, all analyses were done using soybean Williams 82 genome (version 2.0) as references. *Medicago truncatula* genome sequence (version Mt4.0) was downloaded from Phytozome. Short reads from representative soybean accessions were downloaded from GenBank.

### Bioinformatics

DNA reads were mapped to the soybean reference genome by BWA with the BWA-MEM algorithm [87]. R packages *Rsamtools* [88] and *GenomeGraphs* [89] were used to analyze and display the sequence coverage in candidate genes. The missing genotypes in candidate genes were imputed by fastPhase under the context including up- and downstream 20 kb regions [90]. R package *pegas* was used to reconstruct the haplotype networks with SNPs detected in the genes [91]. All the other analyses are done with in-house scripts written in Perl or R. Base-composition across genome-wide SNP sites was calculated as described in a previous study [5]. Because of PR2, i.e., nucleotide A content ([A]) from SNP sites is roughly equals to [T] ([A] ≈ [T]) and [C] ≈ [G] [5], the value of [AT] was used in this study.

### Base-composition distribution among substitution types

Bi-allelic SNPs can be grouped into 6 substitution types (A/C, A/G, A/T, C/G, C/T, and G/T) without a defined ancestral allele. For example, if C and T alleles are detected in 1 SNP site, which might arise either from C to T change or from T to C change, it is a C/T substitution type. For each substitution type, the total number of each nucleotide type possessed by each accession was counted and divided by the total number of polymorphic sites (8.9 million in maize and 4.9 million in soybean for the accession without missing calls).

### Base-composition distribution at different genomic regions

SNP effects were predicted with the SnpEff v4.3 [92]. In maize, we built the database with reference genome sequences available at ftp://ftp.ensemblgenomes.org/pub/plants/release-39/fasta/zea_mays/dna/Zea_mays.AGPv4.dna.toplevel.fa.gz and gene annotation available at ftp://ftp.ensemblgenomes.org/pub/plants/release-39/gff3/zea_mays/Zea_mays.AGPv4.39.chr.gff3.gz. In soybean, we built the database with reference genome sequences available at ftp://ftp.ensemblgenomes.org/pub/plants/release-39/fasta/glycine_max/dna/Glycine_max.Glycine_max_v2.0.dna.toplevel.fa.gz and gene annotation available at ftp://ftp.ensemblgenomes.org/pub/plants/release-39/gff3/glycine_max/Glycine_max.Glycine_max_v2.0.39.chr.gff3.gz.

Seven genomic annotation sets (intergenic, gene-proximal, UTRs, synonymous, missense, intronic, and other genic) were obtained by classifying SNPs based on the predicted SNP effect. SNPs were classified to be gene-proximal if they fell within 5 kb upstream of the transcription start site. Then, intergenic set together with the gene-proximal set is considered as non-genic SNP set, and the rest five SNP sets are considered to be genic SNP set. After that, base-composition across polymorphic sites was calculated for genic SNP set and non-genic SNP set separately.

The physical positions for maize centromeric corresponding to the genome (version AGPv4) were referred from a previous study [93]. Then, a 40-Mb segment directly adjacent upstream and downstream of the centromeric region was considered as pericentromeric regions based on a previous study [33]. And the physical coordinates for soybean centromeric and pericentromeric regions were obtained from [34] and Soybean Genome Browser at SoyBase https://soybase.org/gb2/gbrowse/gmax2.0/.

To analyze the base-composition distribution along chromosomes, we calculated the [AT] for each accession with a moving average approach of a 5-Mb window size and a 4-Mb step size on each of the maize and soybean chromosomes with both genic and non-genic SNPs. Indeed, we examined the [AT] distribution with a series of window size including 1 Mb, 2 Mb, 5 Mb, and 10 MB. The patterns for all of those window sizes are similar. We decided to go with the 5 Mb for the analyses because it contains a good amount of SNPs in each window and the line of [AT] distribution is smoother than the smaller window size.

The position of crossovers (COs) in maize was referred from [39]. Then, [AT]-difference and crossover (CO) rate were calculated using a 5-Mb sliding window. Recombination rate data in soybean was referred from [30]. [AT]-difference and recombination rate were calculated using a 1-Mb window. The correlation was calculated between [AT]-difference and CO rate or recombination rate for each chromosome.

Transposable element (TE) regions in maize and soybean are referred from [93, 94]. Then, base-composition across polymorphic sites was calculated for SNPs within TE regions and non-TE regions separately.

Selective sweep regions in maize and soybean are referred from [29, 30]. Then, base-composition across polymorphic sites was calculated for SNPs within selective sweep and non-selective sweep regions separately.

The maize methylation data was generated from whole-genome bisulfite sequencing (WGBS) of the leaf tissue of maize B73 seedling [42]. Genome coordinates of B73 methylation data in AGPv2 were converted to that in AGPv4 with the CrossMap v0.2.5 [84]. Then, the maize genome was separated into methylated and unmethylated regions based on whether the percentage of CG methylation within each 100 bp non-overlapping window is greater than 40% or not. The soybean methylation data was generated from WGBS of the leaf of soybean Williams 82 [43] and GsojaD [44].

MethylC-seq reads of GsojaD were first mapped to its own genome assembly to get methylation call. Then, the genome coordinates of GsojaD methylation were converted to the coordinates in Williams 82 genome version 2. Genome coordinates of Williams 82 methylation data in Williams 82 version 1.1 were converted to those in version 2.0 with the CrossMap v0.2.5 [84]. Then, the soybean genome was separated into the methylated and unmethylated regions based on CG methylation sites that are common to both Williams 82 and GsojaD.

### Motif enrichment analysis

For each SNP site, the directly adjacent upstream and downstream bases were extracted from reference genomes; meanwhile, the adjacent sequences of 1 randomly selected site from 1 kb flanking region were also extracted. For each of the 96 possible tri-nucleotide motifs (5′-NXN-3′, X is the polymorphic site or randomly selected site), an empirical threshold at the 95th percentile was drawn from 100 random sample scenarios. A motif is considered as enriched if the ratio of its frequency at SNP site over the 95th percentile at random site is greater than 1.

### Population-private SNP analysis

We used the procedure laid out in a previous study [6] to test the mutation spectrum differences between populations with population-private SNPs. SNPs within each private SNP set were partitioned into 96 mutation types through considering the base immediately upstream and downstream of the variable site [47]. Count data *C*_*p*_(*m*) of type *m* mutations in set *P* for each mutation type *m* = ${B}_{5^{\prime }}{B}_A{B}_{3^{\prime }}$ → ${B}_{5^{\prime }}{B}_D{B}_{3^{\prime }}$ of each private SNP set *P* were obtained. Then, with a *χ*^2^ test, *f*_PI_(*m*) and *f*_PL_(*m*) were compared with *f*_PW_(*m*). For the *χ*^2^ test, we used *χ*^2^ value instead of *P* value to indicate the significance of difference because *P* value cannot be obtained for very large *χ*^2^ value in our data.

To assess the variance of *f*(*TCG* → T) and *f*(*CCG* → T), private SNP sets PL, PI, and PW in maize and PD and PW in soybean was partitioned into non-overlapping bins of 1000 consecutive SNPs. Then, *f*(*TCG* → T) and *f*(*CCG* → T) for each bin were calculated.

### GWAS for base composition in maize and soybean

Following our earlier study in human [5], [AT] values across 8,852,678 maize SNPs and 4,870,265 soybean SNPs were used as the genome phenotype for GWAS. In the genome scan for both maize and soybean, a mixed linear model (MLM) with both fixed covariates and a random kinship matrix was used to detect SNPs associated with the base-composition variation [95, 96] in GAPIT version 3.35 [97]. Parameters in MLM were determined by model selection process [95, 96]. Five principle components (PC2-PC6) were selected in maize, and 0 PC was selected in soybean. PC1 was not under the model selection process because of its near-perfect correlation with [AT] [5]. The significance threshold *P* value was determined by Bonferroni correction.

The 334 maize genes and 107 soybean genes associated with repairing UV-damaged DNA were compiled based on either the sequence similarity of rice genes or *Arabidopsis* genes [48]. We conducted enrichment test of UV-related genes with a series of window sizes centered by significantly associated SNPs as described in a previous study [31]. The proportion of UV-related genes within each window was compared with its genome-wide proportion. The gene was counted when it was tagged by at least 2 significantly associated SNPs. Then, we tested whether the proportion of UV-related genes within the window is significantly higher than that across the whole genome using a proportion test. The window size smaller than 500 kb in maize and 200 kb in soybean was not tested because their numbers of tagged UV-related genes were less than 10, which violated the condition of the proportion test.

## Additional file

Additional file 1: **Figure S1.** Diagram of SNP filtering process. **Figure S2.** Frequency of SNP substitution types. **Figure S3.** Base-composition distribution for randomly sampled genic and non-genic SNPs. **Figure S4.** Comparison of base-composition distribution between different regions of the genome. **Figure S5.** Distribution of MAF for genome-wide genic and non-genic SNPs. **Figure S6**-**S8.** Base-composition distribution for genic and non-genic SNPs across chromosomes. **Figure S9.** Base-composition distribution at TE and non-TE regions. **Figure S10.** Base-composition distribution between domesticated and wild accessions and crossover rate for maize chromosomes. **Figure S11**-**S12**. Base-composition distribution between domesticated and wild accessions and recombination rate for soybean chromosomes. **Figure S13**-**S15.** Distribution of MAF calculated with genic and non-genic SNPs across chromosomes. **Figure S16.** Base-composition distribution at selective sweep and non-selective-sweep regions. **Figure S17.** Frequencies of motifs related to solar-UV signature among genic and non-genic SNPs conditional on methylated and unmethylated regions. **Figure S18.** Frequencies of motifs related to solar-UV signature among SNPs from pericentromeric and non-pericentromeric regions under methylated and unmethylated conditions. **Figure S19.** Enrichment test of mutations related to solar-UV signature with population-private SNPs. **Figure S20.** Distribution of *f*(TCG) and *f*(CCG) across population-private SNPs. **Figure S21.** Distribution of *f*(TCG) and *f*(CCG) at different genomic regions. **Figure S22.** GWAS-identified genomic regions underlying base-composition variation. **Figure S23.** GWAS tagged genes in NER pathway. **Figure S24.** Polymorphisms in soybean *DNA ligase1*. **Table S1.** UV-related genes are enriched near the associated loci in maize. **Table S2.** UV-related genes tagged by the associated SNPs in maize. **Table S3.** UV-related genes are enriched near the associated loci in soybean. **Table S4.** UV-related genes tagged by the associated SNPs in soybean. **Table S5.** Summary of 100 maize accessions. **Table S6.** Summary of 302 soybean accessions. (PDF 3092 kb)

## Abbreviations

CO: Crossover
CPDs: Cyclobutane pyrimidine dimers
DCH: Domesticated cluster haplotype
DMRs: Differentially methylated regions
GWAS: Genome-wide association studies
MAF: Minor allele frequency
NER: Nucleotide excision repair
PD: Private domesticated SNPs
PI: Private improved cultivar SNPs
PL: Private landrace SNPs
PW: Private wild SNPs
TE: Transposable element
UV: Ultraviolet
WGBS: Whole-genome bisulfite sequencing
