# Supplementary material for: Genome-wide nucleotide patterns and potential mechanisms of genome divergence following domestication in maize and soybean
Source: Genome Biol. 2019 Apr 25;20:74. doi: 10.1186/s13059-019-1683-6 (PMC6482504; doi:10.1186/s13059-019-1683-6)
Supplement: Supplementary file 1 — Figure S1. Diagram of SNP filtering process. Figure S2. Frequency of SNP substitution types. Figure S3. Base-composition distribution for randomly sampled genic and non-genic SNPs. Figure S4. Comparison of base-composition distribution between different regions of the genome. Figure S5. Distribution of MAF for genome-wide genic and non-genic SNPs. Figure S6-S8. Base-composition distribution for genic and non-genic SNPs across chromosomes. Figure S9. Base-composition distribution at TE and non-TE regions. Figure S10. Base-composition distribution between domesticated and wild accessions and crossover rate for maize chromosomes. Figure S11-S12. Base-composition distribution between domesticated and wild accessions and recombination rate for soybean chromosomes. Figure S13-S15. Distribution of MAF calculated with genic and non-genic SNPs across chromosomes. Figure S16. Base-composition distribution at selective sweep and non-selective-sweep regions. Figure S17. Frequencies of motifs related to solar-UV signature among genic and non-genic SNPs conditional on methylated and unmethylated regions. Figure S18. Frequencies of motifs related to solar-UV signature among SNPs from pericentromeric and non-pericentromeric regions under methylated and unmethylated conditions. Figure S19. Enrichment test of mutations related to solar-UV signature with population-private SNPs. Figure S20. Distribution of f(TCG) and f(CCG) across population-private SNPs. Figure S21. Distribution of f(TCG) and f(CCG) at different genomic regions. Figure S22. GWAS-identified genomic regions underlying base-composition variation. Figure S23. GWAS tagged genes in NER pathway. Figure S24. Polymorphisms in soybean DNA ligase1. Table S1. UV-related genes are enriched near the associated loci in maize. Table S2. UV-related genes tagged by the associated SNPs in maize. Table S3. UV-related genes are enriched near the associated loci in soybean. Table S4. UV-related genes tagged by the associated SNPs in soybe [file 13059_2019_1683_MOESM1_ESM.pdf]

Genome-wide nucleotide patterns and potential mechanisms of genome divergence following domestication in maize and soybean

Jinyu Wang[1], Xianran Li[1*], Kyung Do Kim[2], Michael J. Scanlon[3], Scott A. Jackson[2], Nathan M. Springer[4], Jianming Yu[1*]
[1]Department of Agronomy, Iowa State University, Ames, IA 50011, USA
[2]Center for Applied Genetic Technologies, University of Georgia, Athens, GA 30602, USA
[3]Plant Biology Section, School of Integrative Plant Science, Cornell University, Ithaca, NY 14853, USA
[4]Department of Plant and Microbial Biology, University of Minnesota, St. Paul, MN 55108, USA
[*]Correspondence should be addressed to X.L. (lixr@iastate.edu); J.Y. (jmyu@iastate.edu)

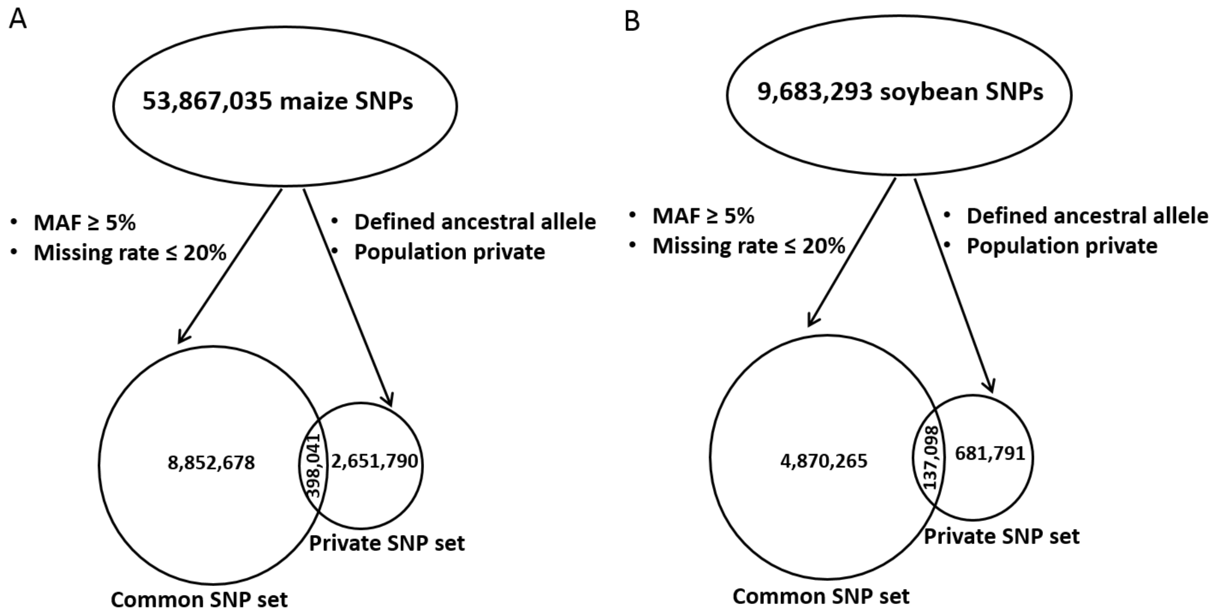

Fig. S1. Diagram of SNP filtering process. (**A**) maize. (**B**) soybean. The common SNP set is filtered with a minor allele frequency (MAF) threshold of 5% and a missing rate threshold of 20%. The private SNP set is obtained by identifying SNPs that have defined ancestral allele information and meet the population-private SNP definition.

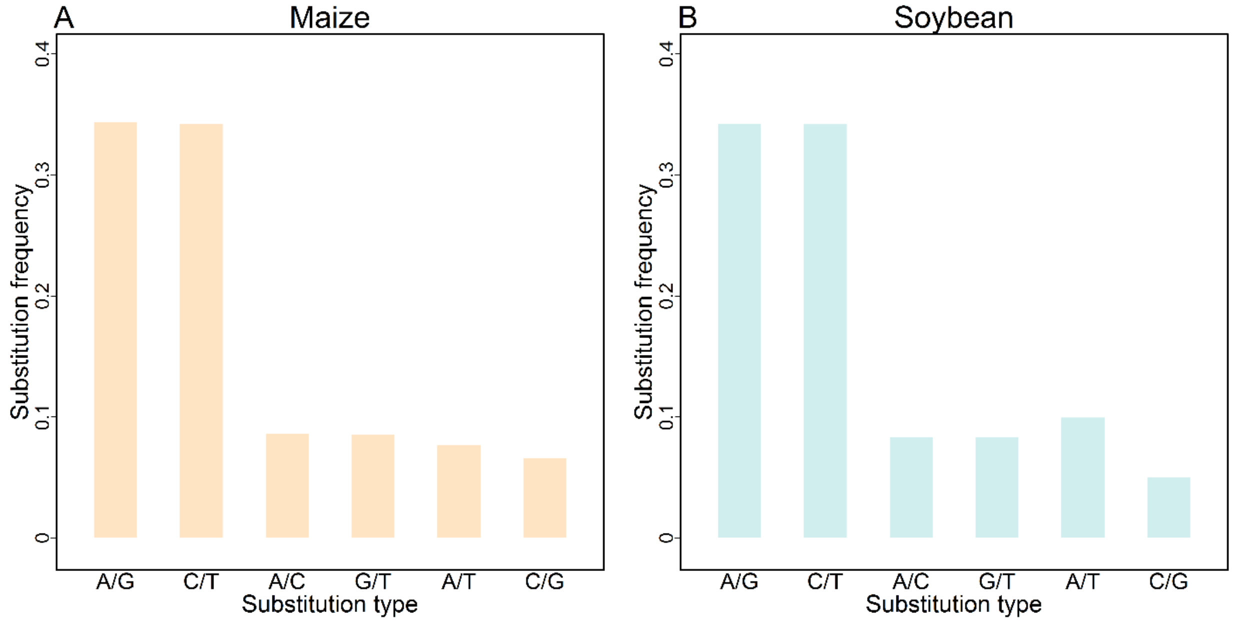

Fig. S2. Frequency of SNP substitution types. (**A**) maize. (**B**) soybean. The genome-wide SNPs were classified into 6 substitution types and frequency for each of the 6 substitution types was calculated.

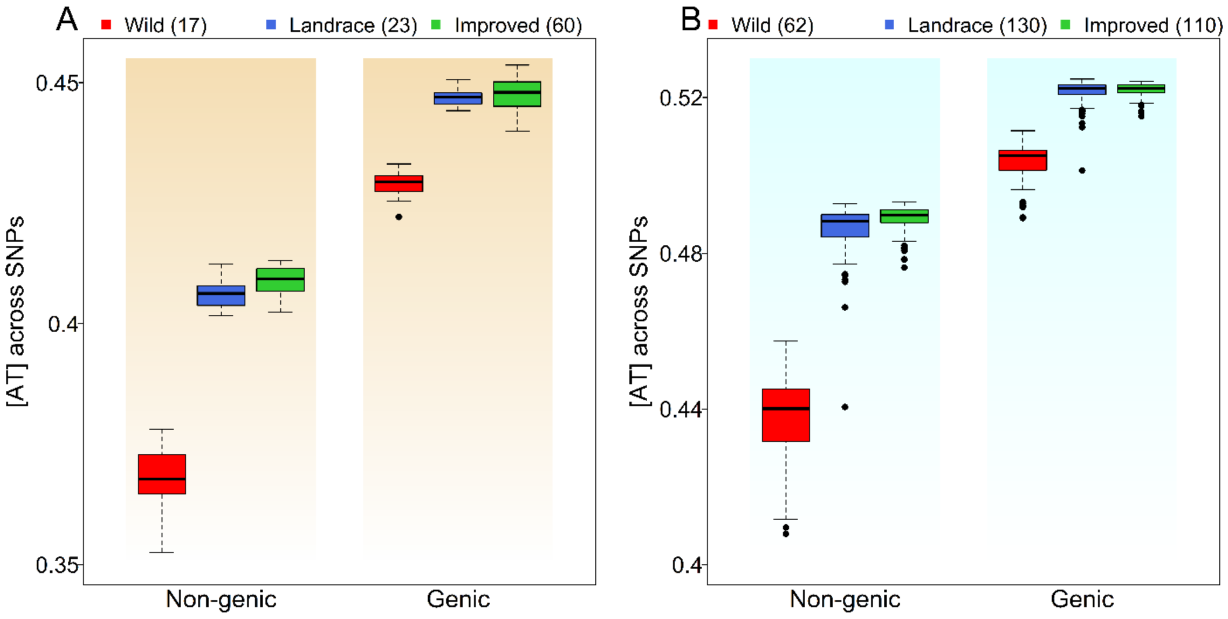

Fig. S3. Base-composition distribution for randomly sampled genic and non-genic SNPs. (**A**) Distribution of [AT] calculated with 1.56 million non-genic SNPs and 1.56 million genic SNPs in maize. (**B**) Distribution of [AT] calculated with 0.7 million non-genic SNPs and 0.7 million genic SNPs in soybean. An equal number of SNPs were randomly sampled from genic and non-genic SNP sets. The average [AT] values over 100 iterations were plotted.

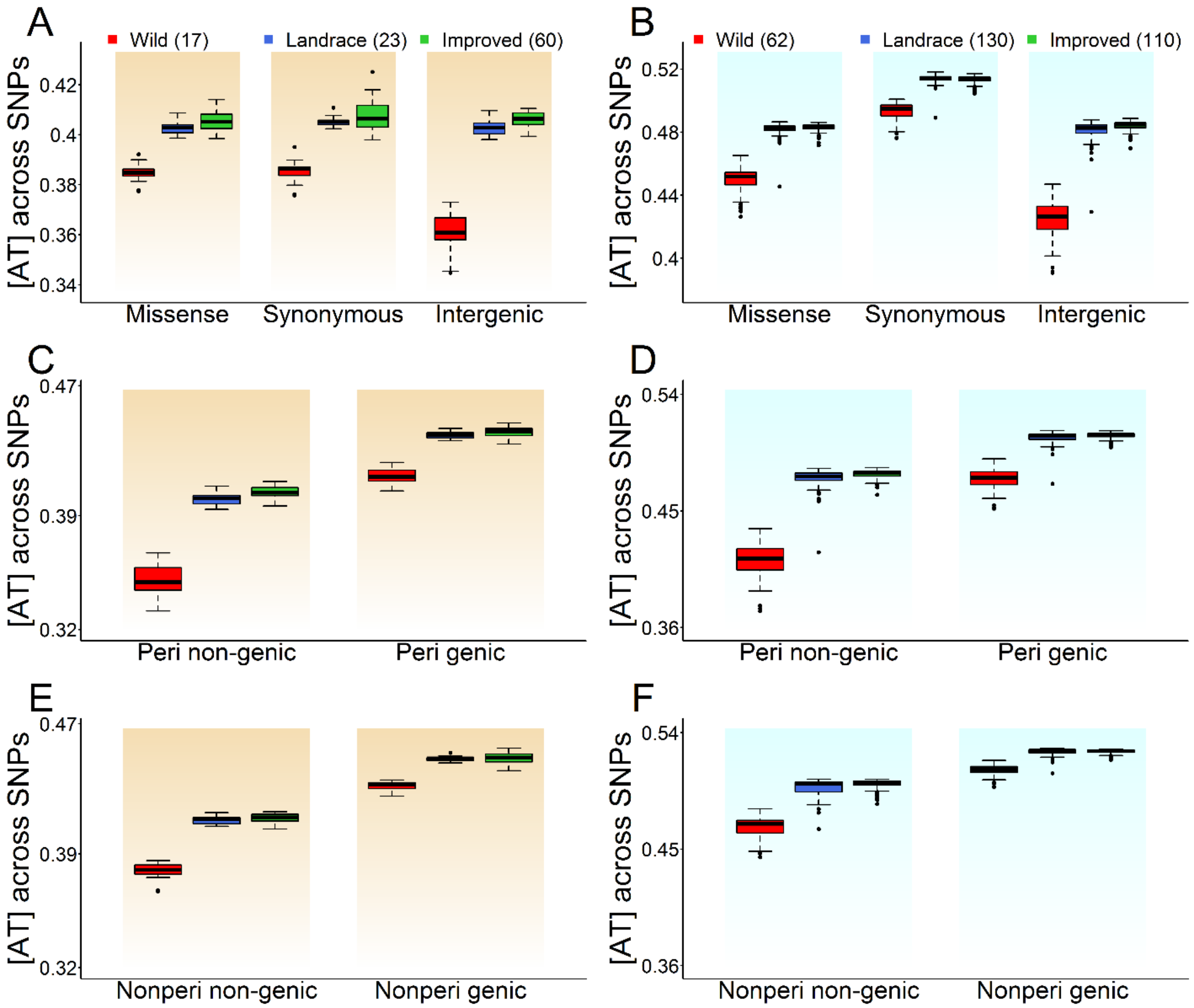

Fig. S4. Comparison of base-composition distribution between different regions of the genome. (**A**) Comparison between missense, synonymous, and intergenic SNP sets in maize. (**B**) Comparison between missense, synonymous, and intergenic SNP sets in soybean. (**C**) Comparison between genic and non-genic regions conditional on pericentromeric regions in maize. (**D**) Comparison between genic and non-genic regions conditional on pericentromeric regions in soybean. (**E**) Comparison between genic and non-genic regions conditional on chromosome arms in maize. (**F**) Comparison between genic and non-genic regions conditional on chromosome arms in soybean.

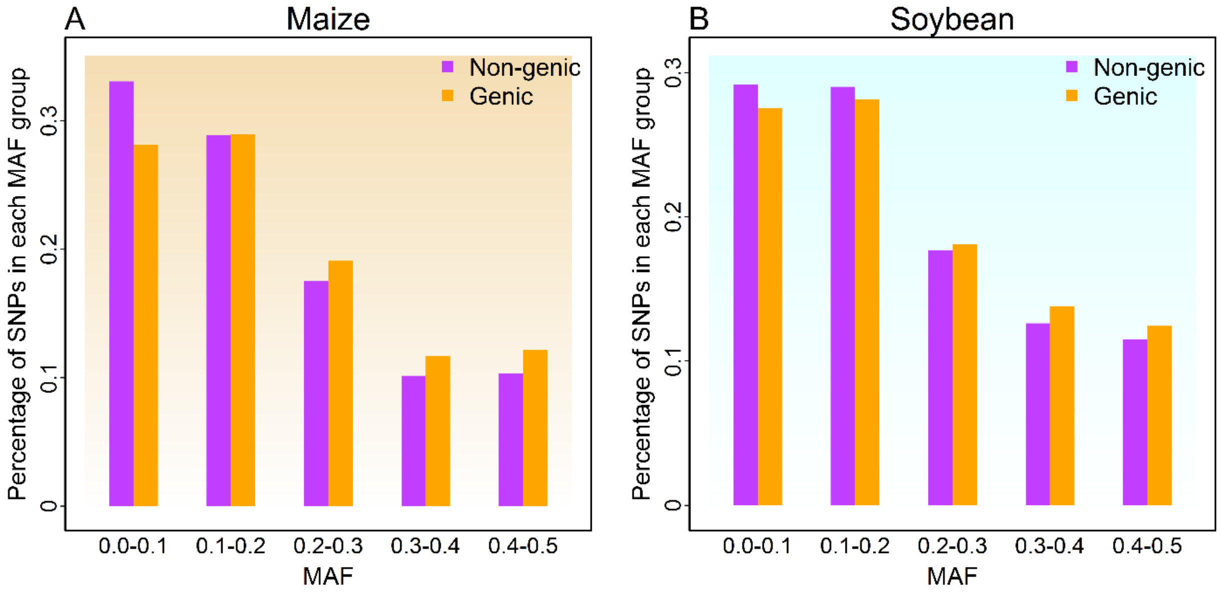

Fig. S5. Distribution of MAF for genome-wide genic and non-genic SNPs. (**A**) maize. (**B**) soybean. The percentage of SNPs within each MAF bin was calculated for genic and non-genic SNPs separately.

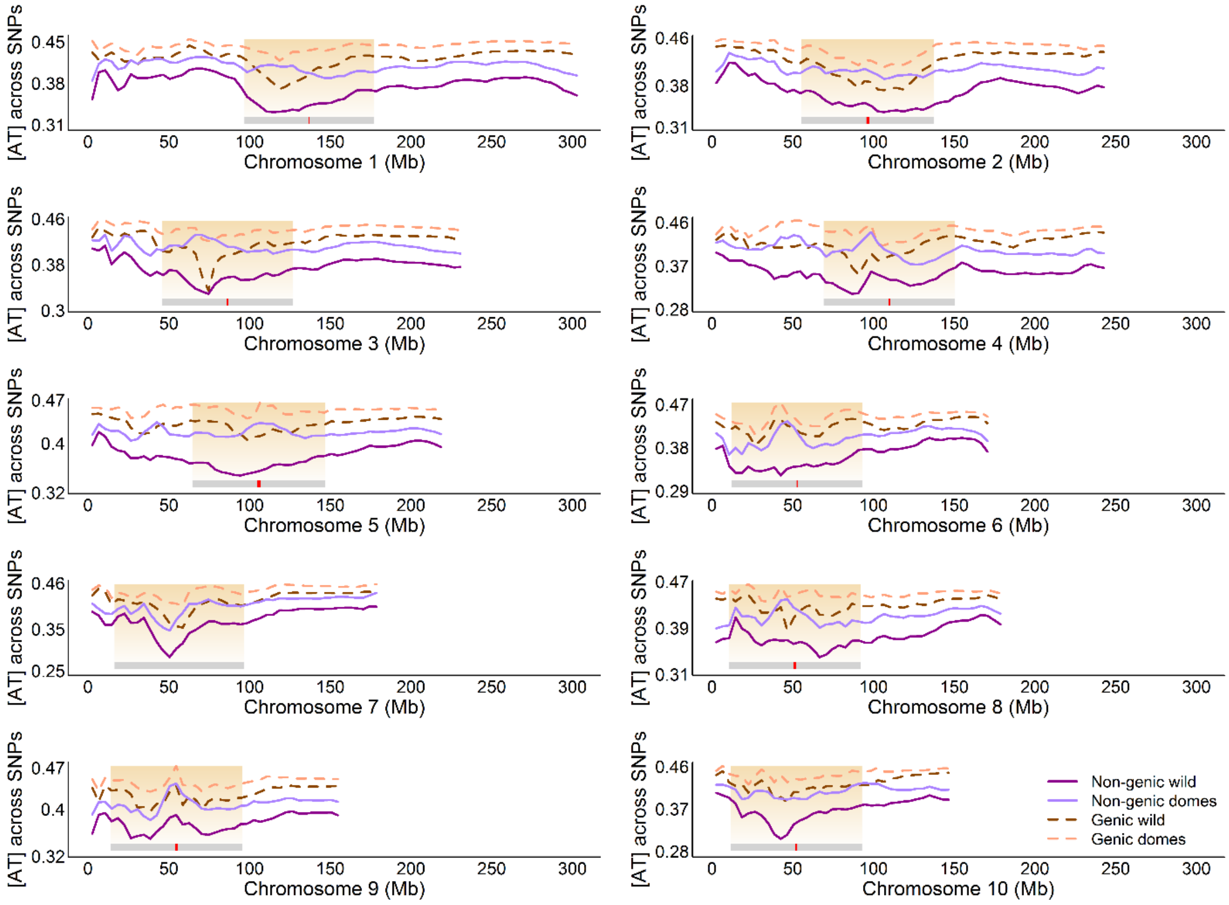

Fig. S6. Base-composition distribution for genic and non-genic SNPs across maize chromosomes. Landraces and improved cultivars are combined to be domesticated group to compare with wild group. For each accession, base-composition was calculated using a moving average approach with a 5-Mb window size and a 4-Mb step size. Each point in the plot represents the mean [AT] of the specified group across a 5-Mb window. The gray bar in the bottom indicates the position of pericentromeric region, and the red bar within gray bar shows the position of centromeric region.

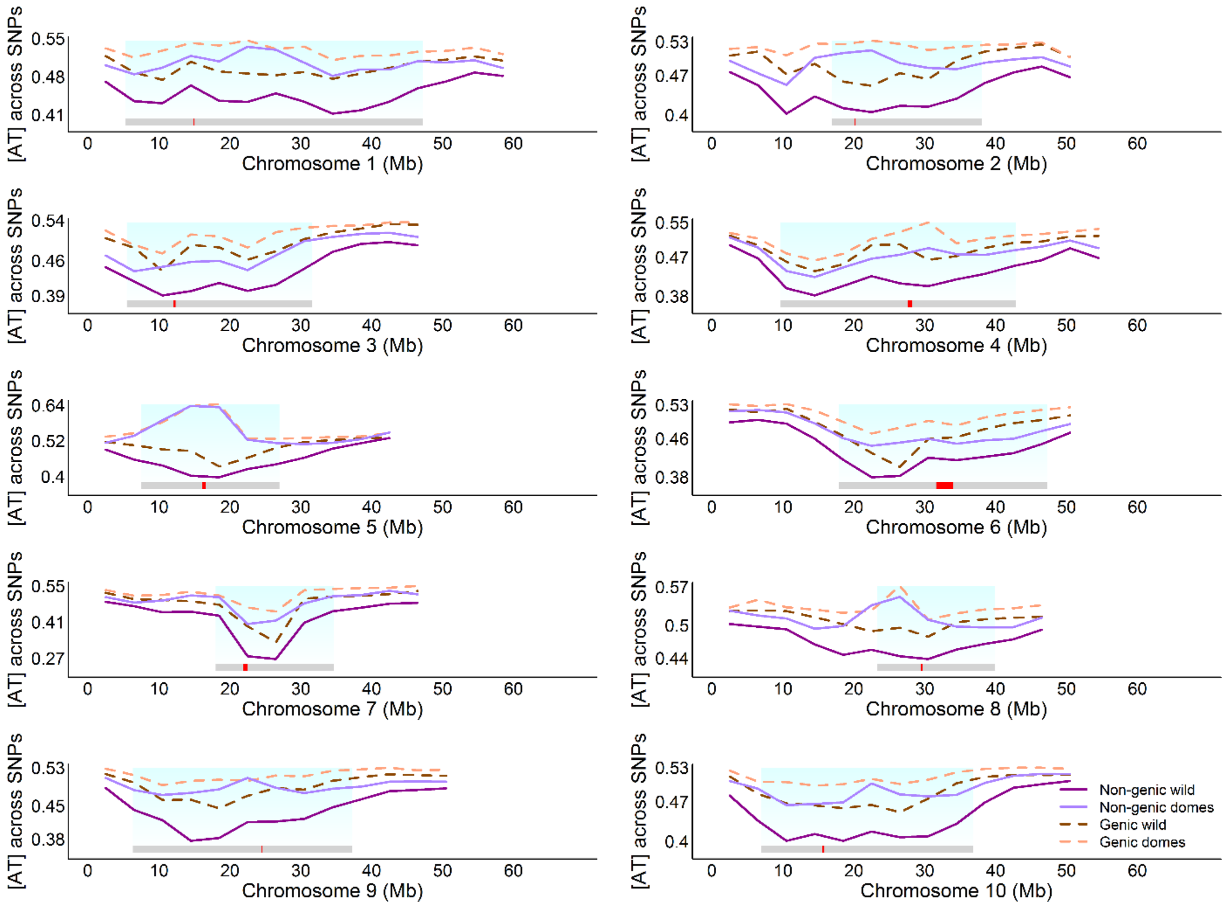

Fig. S7. Base-composition distribution for genic and non-genic SNPs across soybean chromosome 1-10. Landraces and improved cultivars are combined to be domesticated group to compare with wild group. For each accession, base-composition was calculated using a moving average approach with a 5-Mb window size and a 4-Mb step size. Each point in the plot represents the mean [AT] of the specified group across a 5-Mb window. The gray bar in the bottom indicates the position of pericentromeric region, and the red bar within gray bar shows the position of centromeric region. The high [AT] at pericentromeric region (10-20 Mb) of chromosome 5 for domesticated accessions (and the resulting larger [AT]-difference compared with wild accessions) may be due to an extensive selective sweep region detected in this region (Nature Biotechnology 2015, 33:408-414).

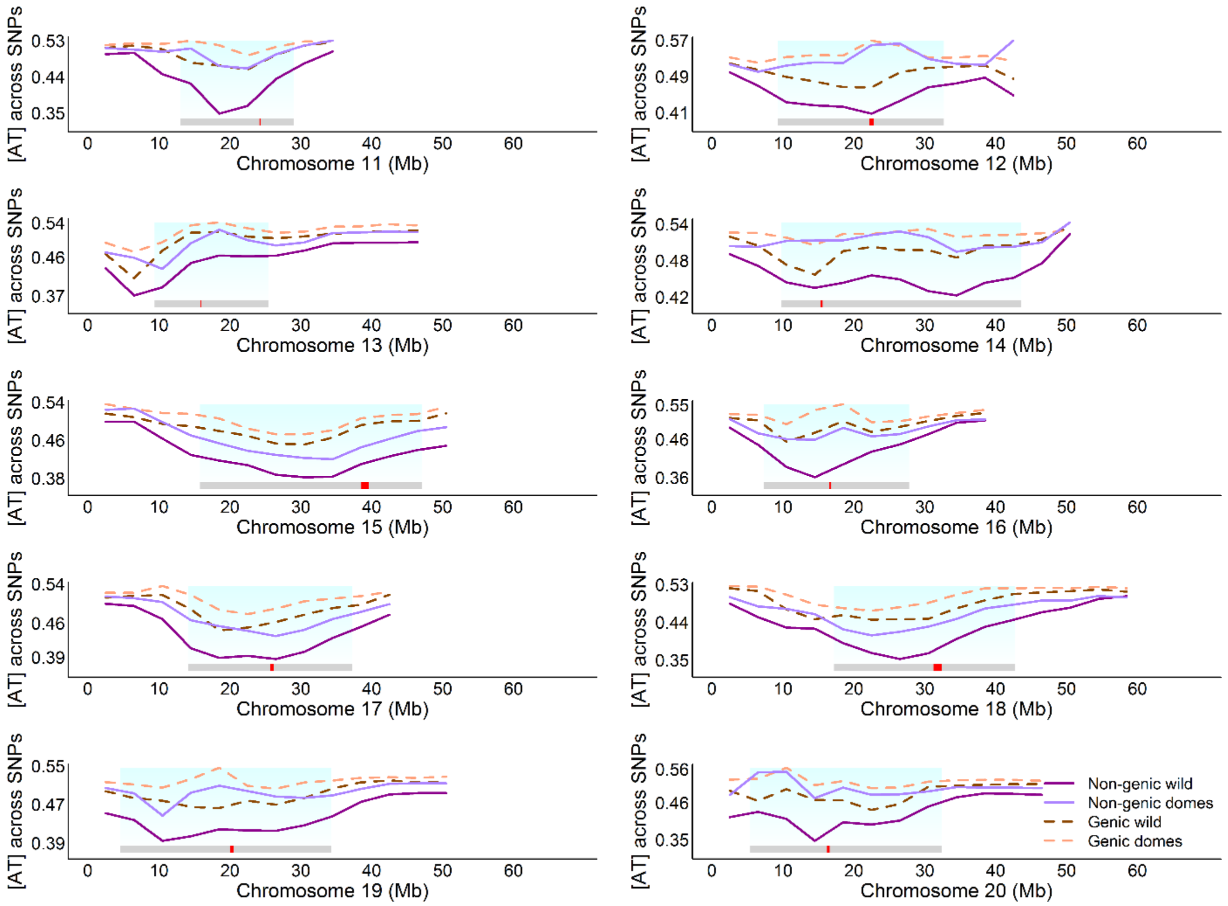

Fig. S8. Base-composition distribution for genic and non-genic SNPs across soybean chromosome 11-20. Landraces and improved cultivars are combined to be domesticated group to compare with wild group. For each accession, base-composition was calculated using a moving average approach with a 5-Mb window size and a 4-Mb step size. Each point in the plot represents the mean [AT] of the specified group across a 5-Mb window. The gray bar in the bottom indicates the position of pericentromeric region, and the red bar within gray bar shows the position of centromeric region.

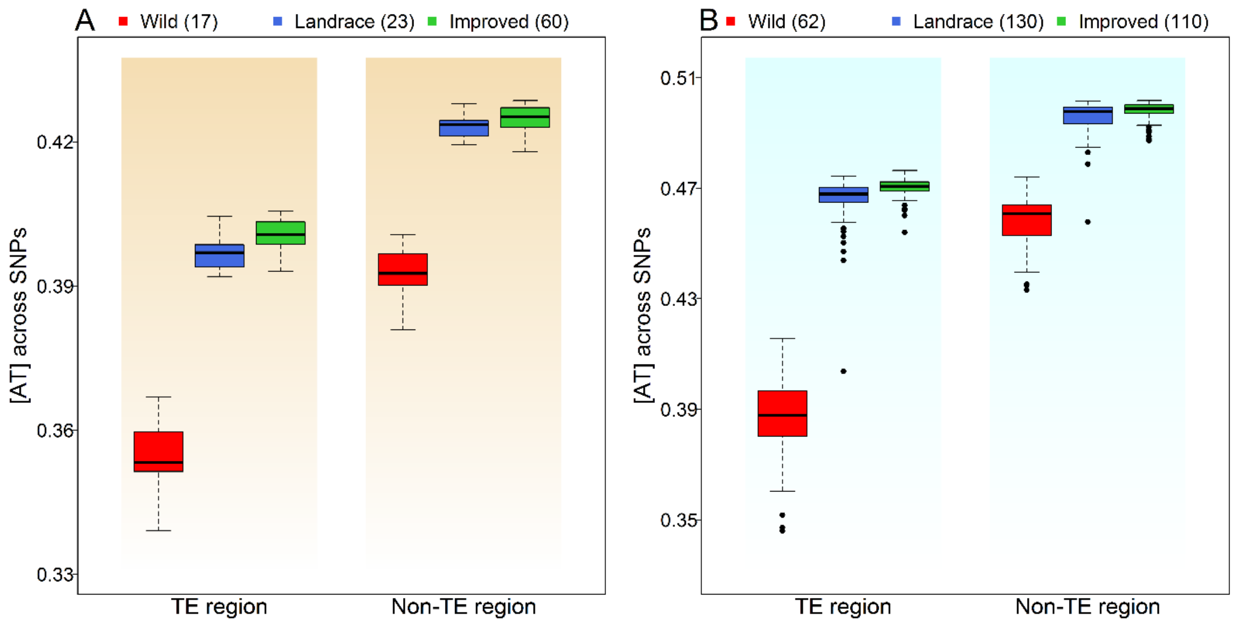

Fig. S9. Base-composition distribution at TE and non-TE regions. (**A**) maize. (**B**) maize. The genome-wide SNPs were classified into TE and non-TE regions to obtain the [AT] values.

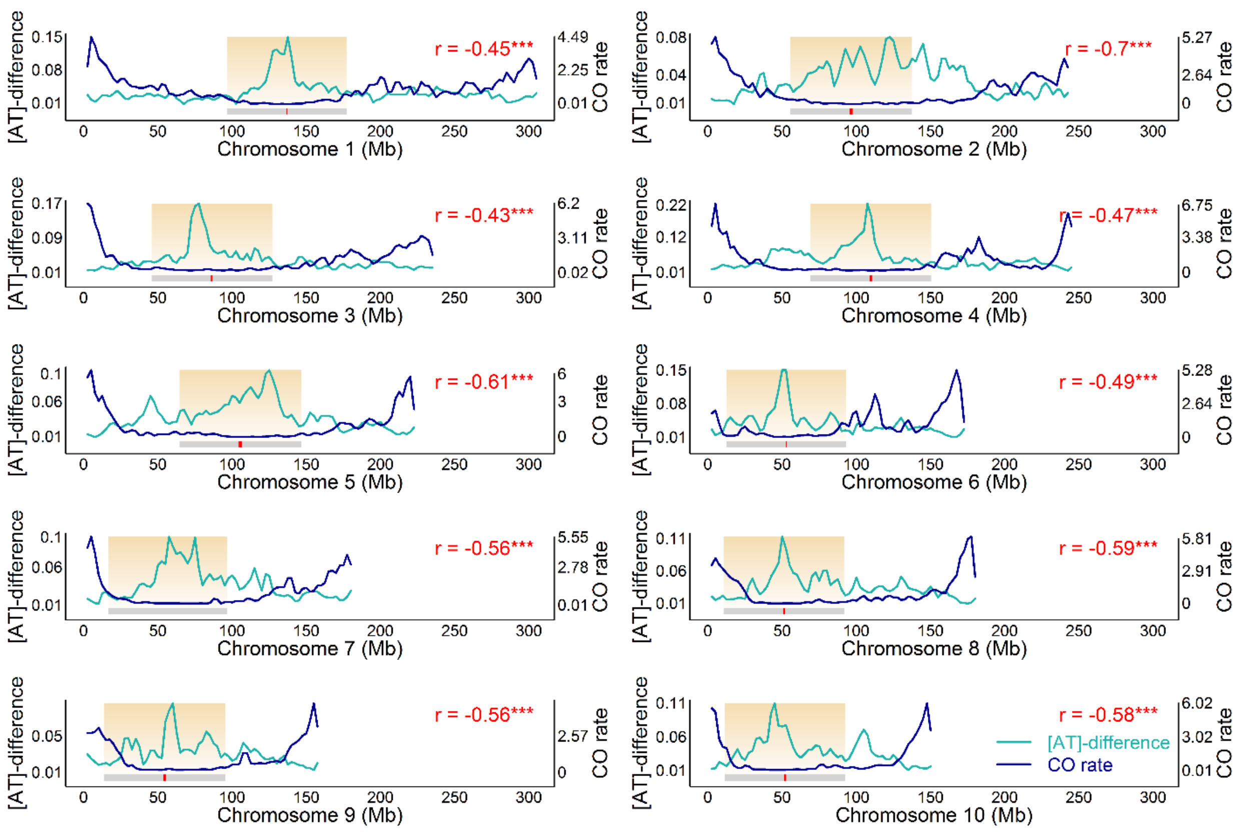

Fig. S10. Base-composition distribution between domesticated and wild accessions and crossover rate for maize chromosomes. Both [AT]-difference CO rate are calculated using a 5-Mb sliding window. *r*, Pearson correlation coefficient between [AT]-difference and CO rate for each chromosome; *, *P*-value ≤ 0.05; **, *P*-value ≤ 0.01, ***, *P*-value ≤ 0.001.

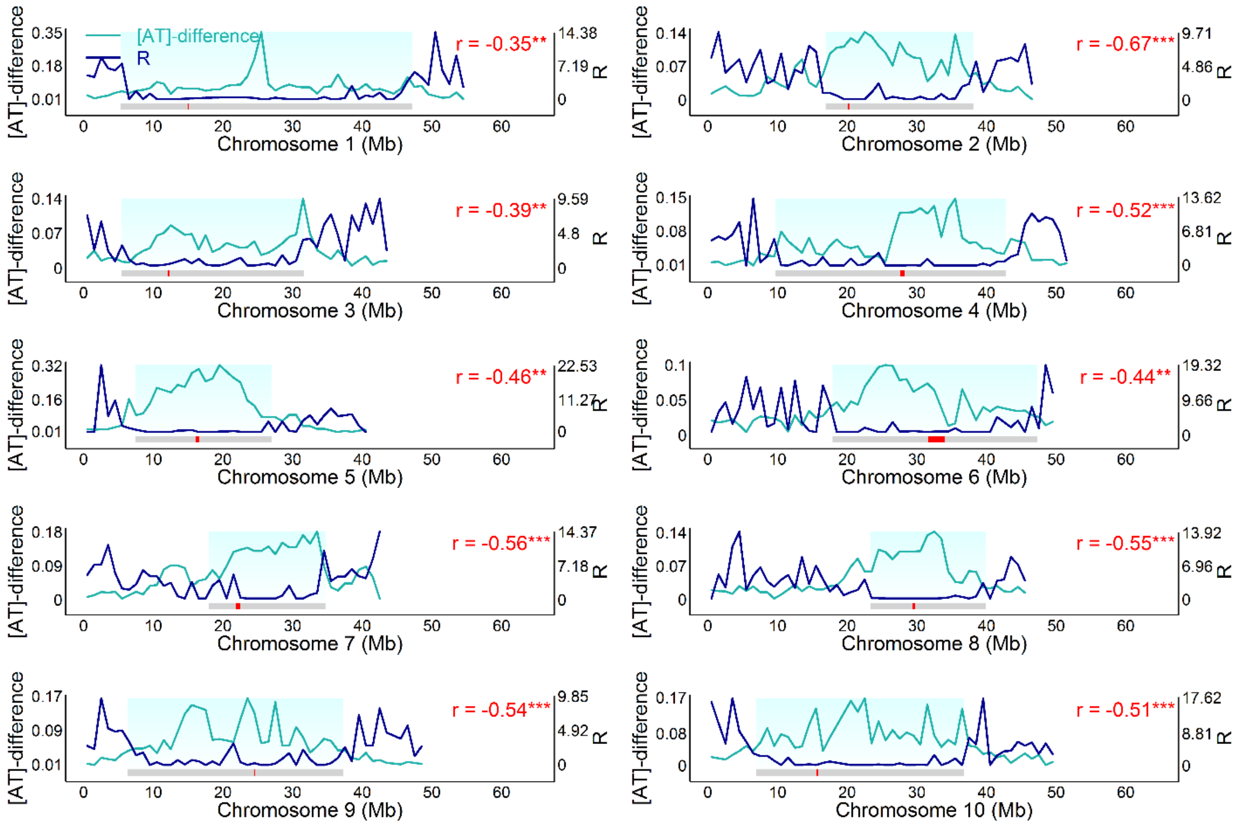

Fig. S11. Base-composition distribution between domesticated and wild accessions and recombination rate for soybean chromosome 1-10. Both [AT]-difference and recombination rate are calculated using a 1-Mb window. *r*, Pearson correlation coefficient between [AT]-difference and recombination rate for each chromosome; *, *P*-value ≤ 0.05; **, *P*-value ≤ 0.01, ***, *P*-value ≤ 0.001.

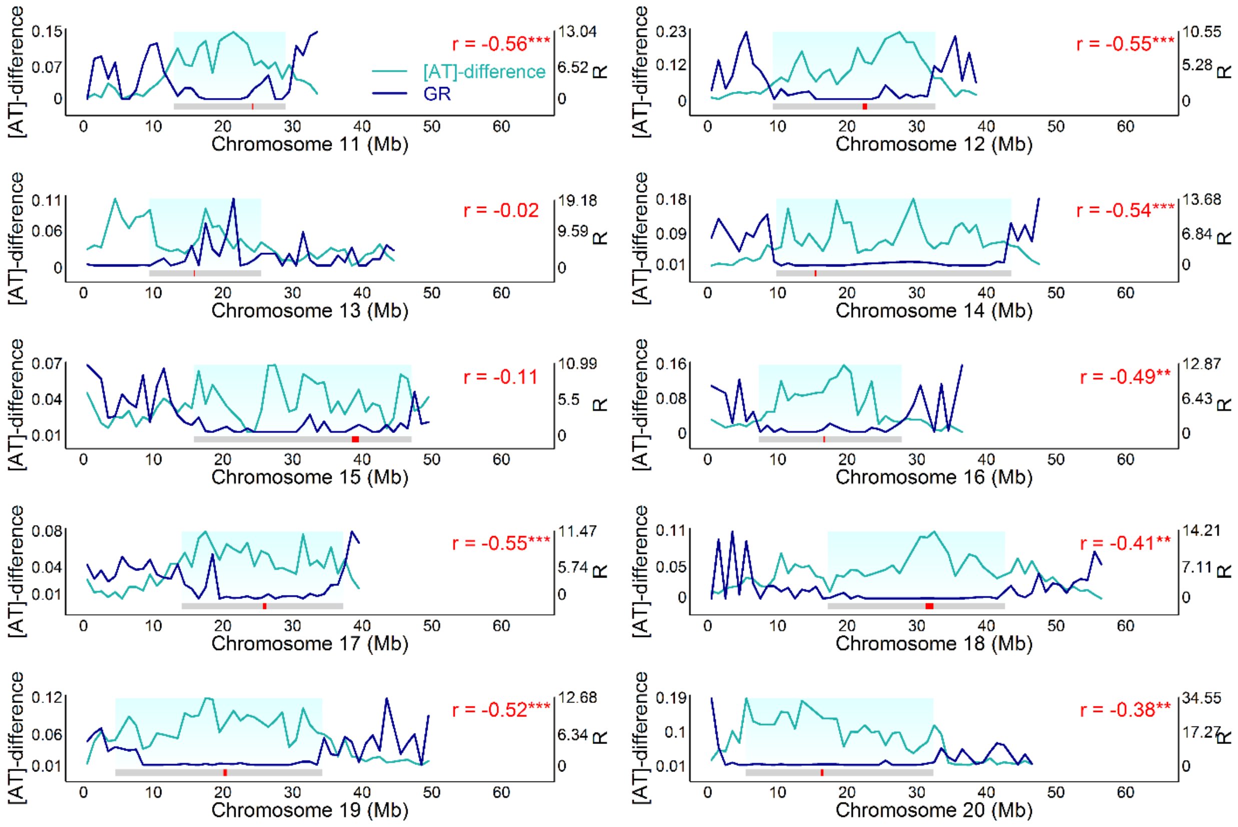

Fig. S12. Base-composition distribution between domesticated and wild accessions and recombination rate for soybean chromosome 11-20. Both [AT]-difference and recombination rate are calculated using a 1-Mb window. *r*, Pearson correlation coefficient between [AT]-difference and recombination rate for each chromosome; *, *P*-value ≤ 0.05; **, *P*-value ≤ 0.01, ***, *P*-value ≤ 0.001.

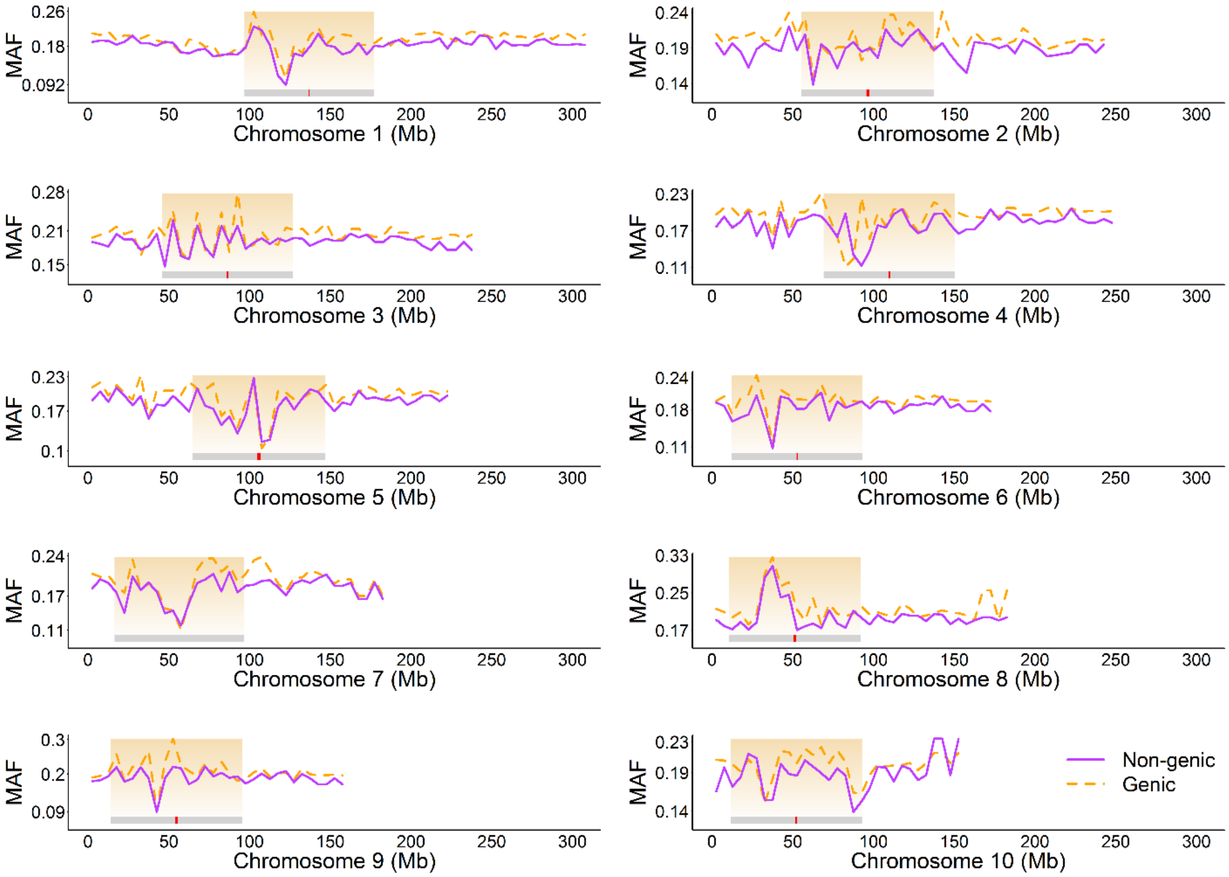

Fig. S13. Distribution of MAF calculated with genic and non-genic SNPs across maize chromosomes. The mean MAF of SNPs was calculated using a moving average approach with a 5-Mb window size and a 4-Mb step size. The gray bar in the bottom indicates the position of pericentromeric region, and the red bar within gray bar shows the position of centromeric region.

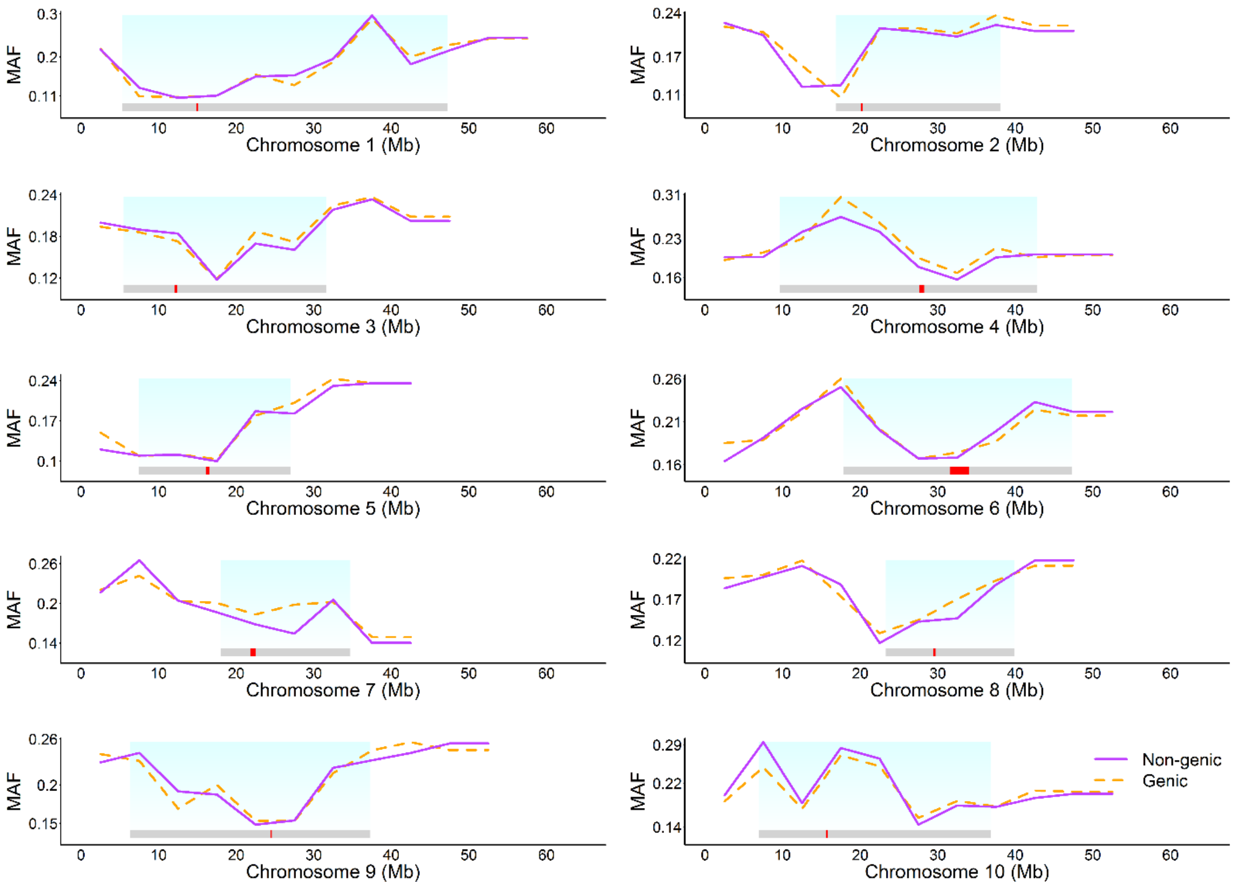

Fig. S14. Distribution of MAF calculated with genic and non-genic SNPs across chromosome 1-10. The mean MAF of SNPs was calculated using a moving average approach with a 5-Mb window size and a 4-Mb step size. The gray bar in the bottom indicates the position of pericentromeric region, and the red bar within gray bar shows the position of centromeric region.

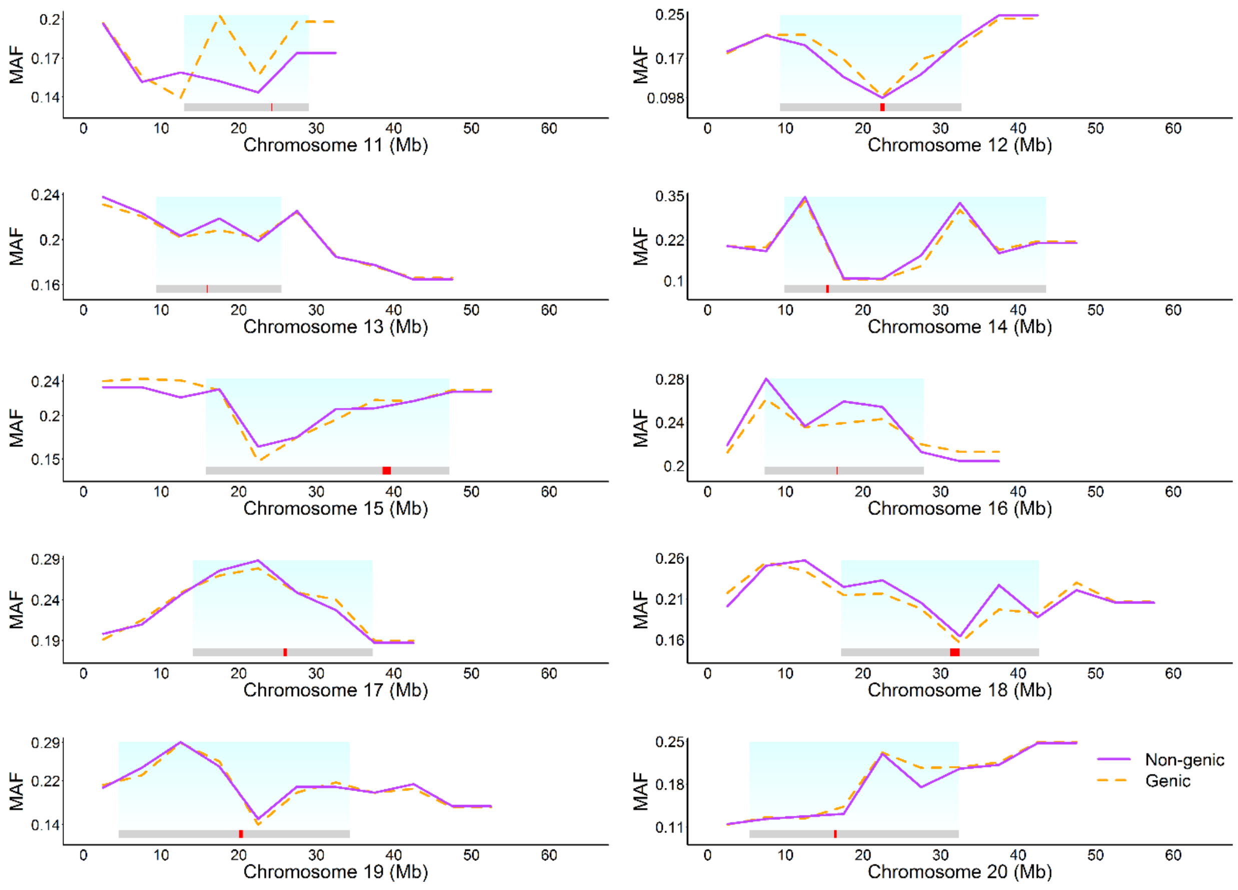

Fig. S15. Distribution of MAF calculated with genic and non-genic SNPs across chromosome 11-20. The mean MAF of SNPs was calculated using a moving average approach with a 5-Mb window size and a 4-Mb step size. The gray bar in the bottom indicates the position of pericentromeric region, and the red bar within gray bar shows the position of centromeric region.

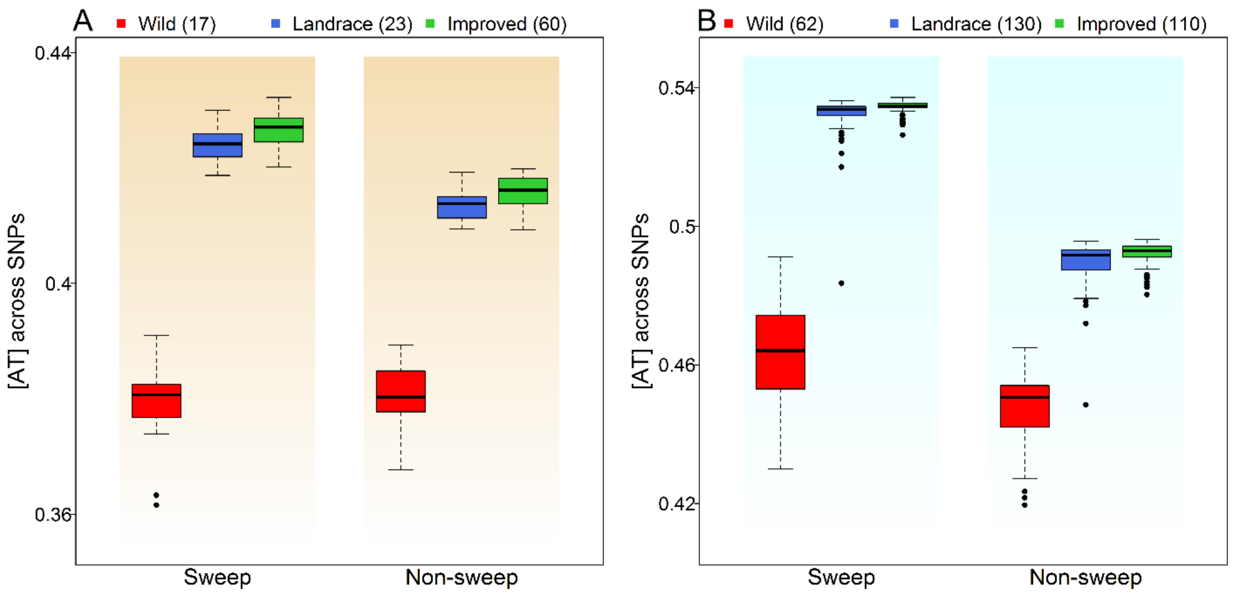

Fig. S16. Base-composition distribution at selective sweep and non-selective-sweep regions in maize (**A**) and soybean (**B**). The genome-wide SNPs were classified into selective sweep and non-selective-sweep regions to calculate [AT] values.

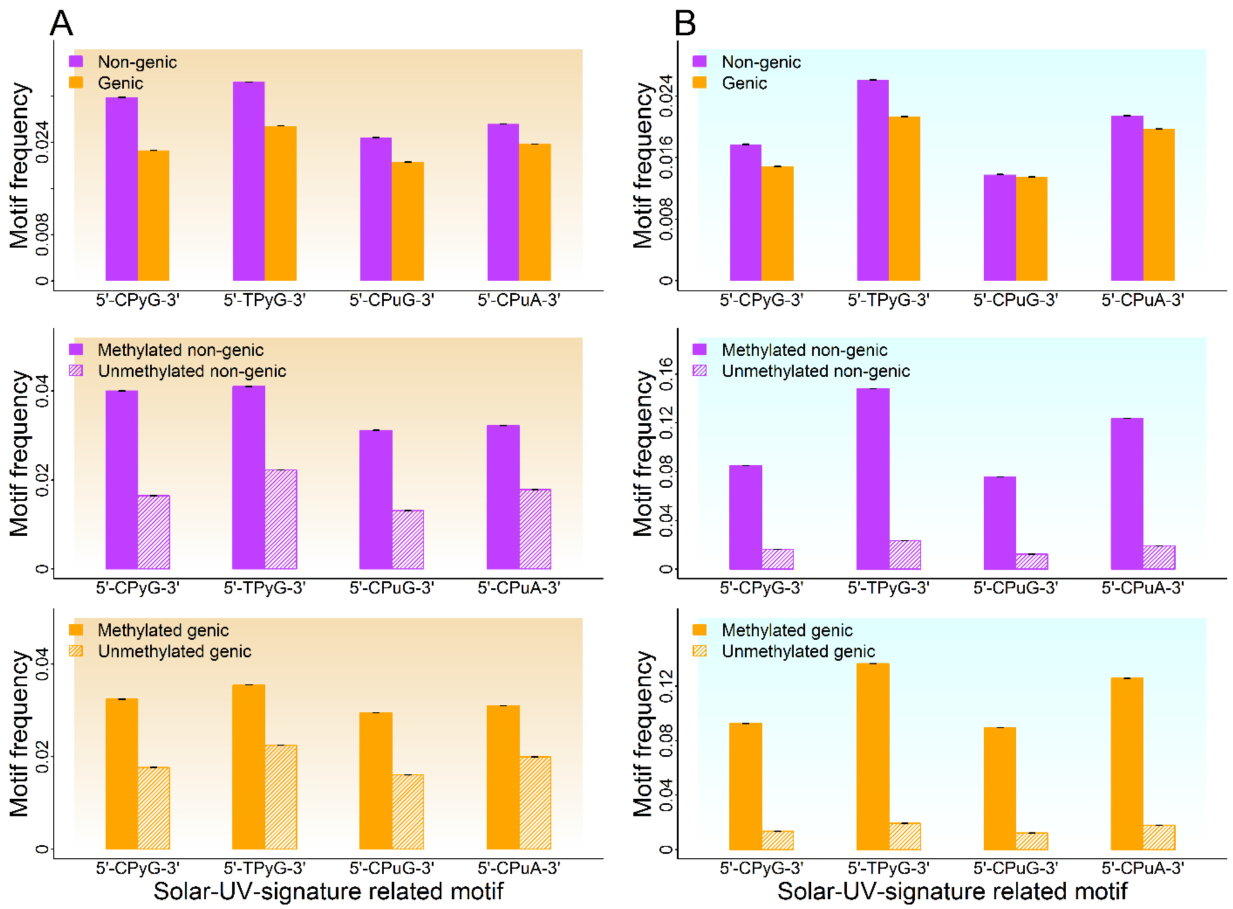

Fig. S17. Frequencies of motifs related to solar-UV signature among genic and non-genic SNPs conditional on methylated and unmethylated regions. (**A**) maize. (**B**) soybean. The top panel shows the frequencies of motifs with all genic and non-genic SNPs. The middle panel shows the frequencies of motifs with non-genic SNPs conditional on methylated and unmethylated regions. The bottom panel shows the frequencies of motifs with genic SNPs conditional on methylated and unmethylated regions. Each bar represents the the average frequency of a specific motif over 100 maize accessions in (**A**) and 302 soybean accessions in (**B**). The black bar on the top illustrates the standardard error.

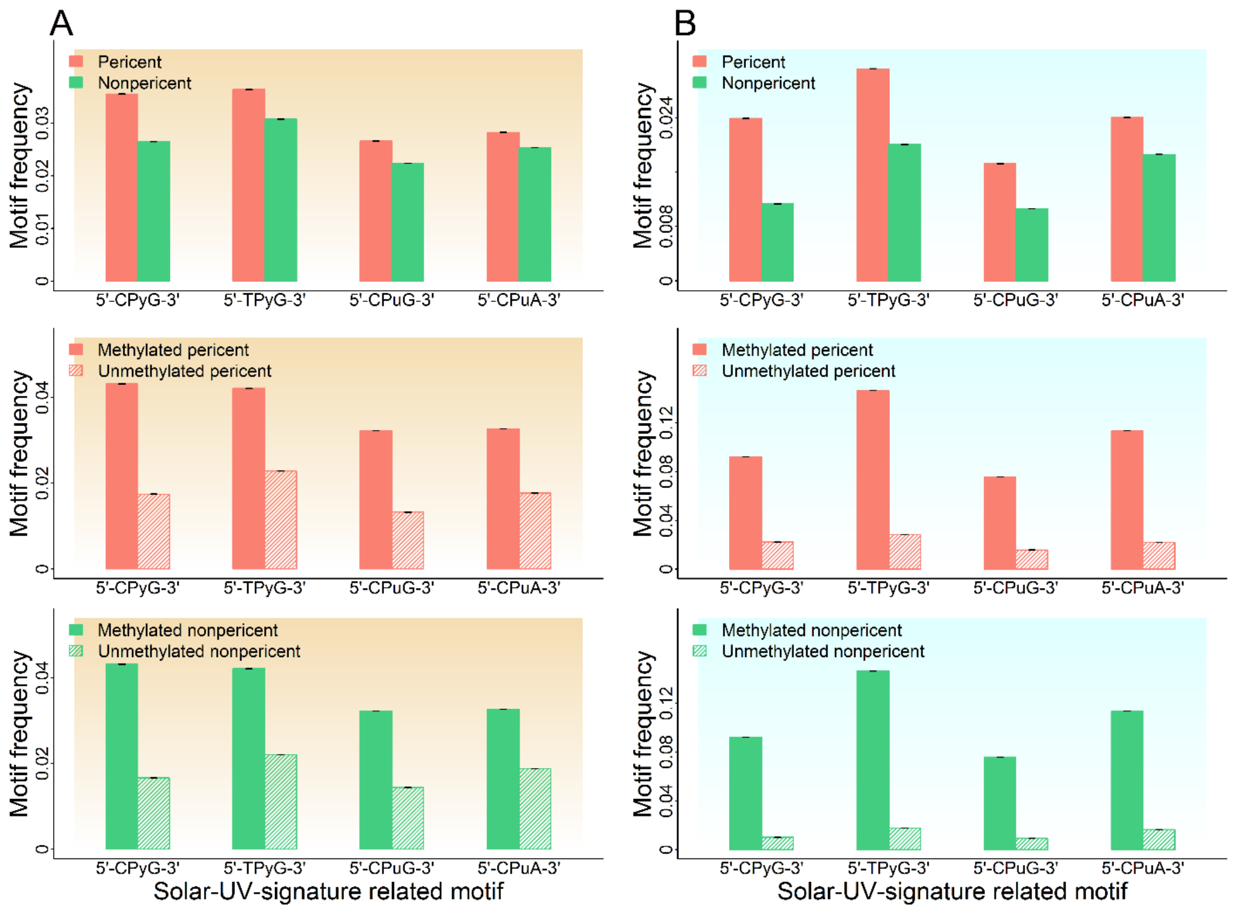

Fig. S18. Frequencies of motifs related to solar-UV signature among SNPs from pericentromeric and non-pericentromeric regions under methylated and unmethylated conditions. (**A**) maize. (**B**) soybean. The top panel shows the frequencies of motifs with SNPs from pericentromeric regions and SNPs from non-pericentromeric regions. The middle panel shows the frequencies of motifs with SNPs from pericentromeric regions conditional on methylated and unmethylated regions. The bottom panel shows the frequencies of motifs with SNPs from non-pericentromeric regions conditonal on methylated and unmethylated regions. Each bar represents the average frequency of a specific motif over 100 maize accessions in (**A**) and 302 soybean accessions in (**B**). The black bar on the top illustrates the standardard error.

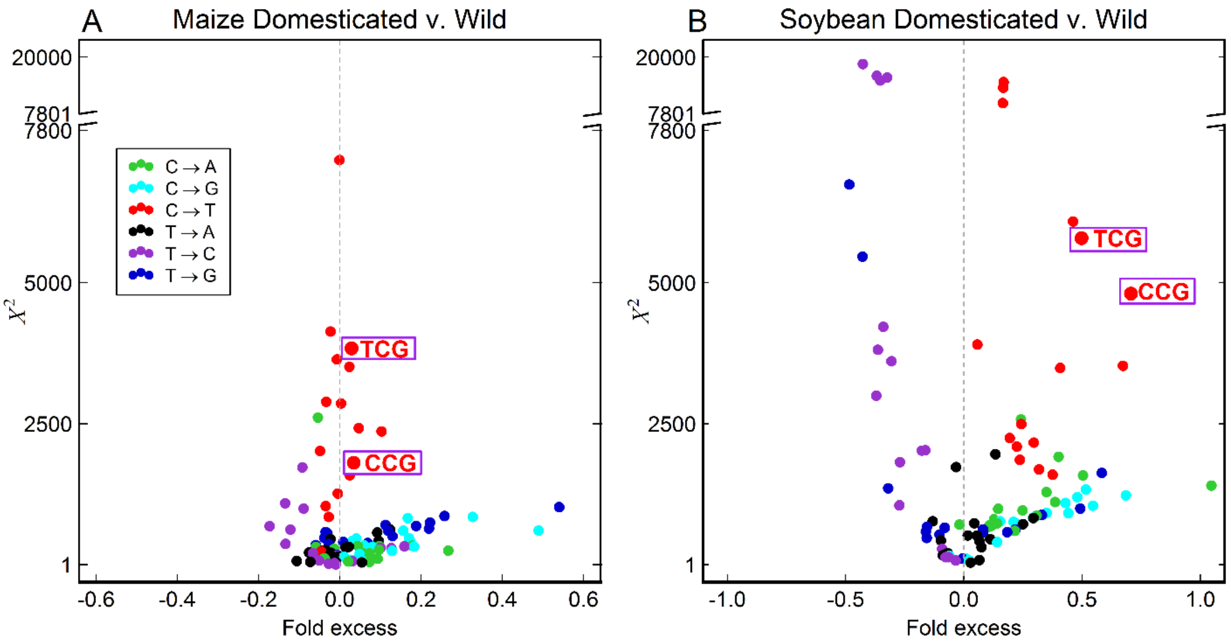

Fig. S19. Enrichment test of mutations related to solar-UV signature with population-private SNPs. Compare the mutation frequency between domesticated accessions and wild accessions in (**A**) maize and (**B**) soybean. The x coordinate of each point indicates the fold frequency difference $(f_{PD}(m) - f_{PW}(m))/f_{PW}(m)$. The y coordinate indicates the Pearson's χ^2 value that measures the significance of the difference between $f_m(P_1)$ and $f_m(P_2)$. Outlier points are labeled with the ancestral state of the mutant nucleotide flanked by two neighboring bases, and the color of the points indicate the ancestral and derived alleles of the mutant site. The purple rectangle highlights the mutations related to solar-UV signature. Here TCG on the plot represents mutation 5′-TCG-3′→5′-TTG-3′ and its reverse complement 5′-CGA-3′→5′-CAA-3′, CCG represents mutation 5′-CCG-3′→5′-CTG-3′ and its reverse complement 5′-CGG-3′→5′-CAG-3′, and similarly for all the other dots on the plot.

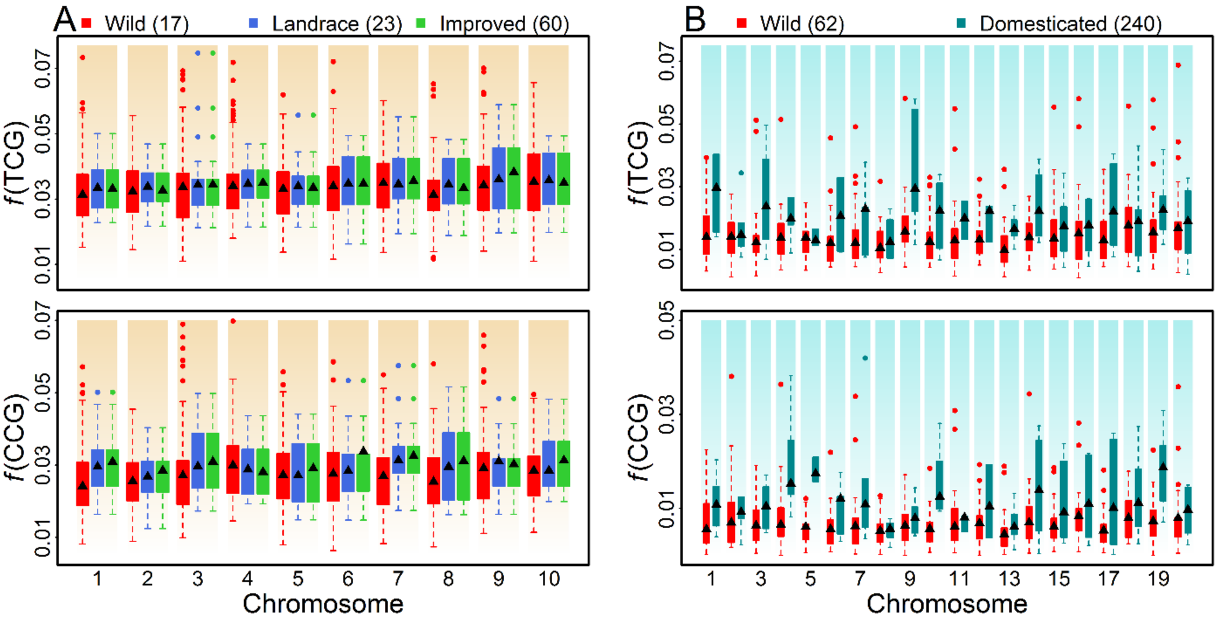

Fig. S20. Distribution of $f(\mathrm{TCG})$ and $f(\mathrm{CCG})$ across population-private SNPs. (**A**) In maize, private SNP sets PW, PL and PI were analyzed. (**B**) In soybean, because of the small number of private SNPs in PL and PI, private SNP sets PD and PW were analyzed. Each private SNP set was partitioned into 1,000 consecutive SNP bins on each chromosome that are not overlapped with each other. The frequency $f(\mathrm{TCG})$ for TCG→T mutation (5′-TCG-3′→5′-TTG-3′ and its reverse complement 5′-CGA-3′→5′-CAA-3′), and the frequency $f(\mathrm{CCG})$ for CCG→T mutation (5′-CCG-3′→5′-CTG-3′ and its reverse complement 5′-CGG-3′→5′-CAG-3′) of each bin were calculated and plotted. The black triangle within each box plot indicates the chromosome-wide frequency.

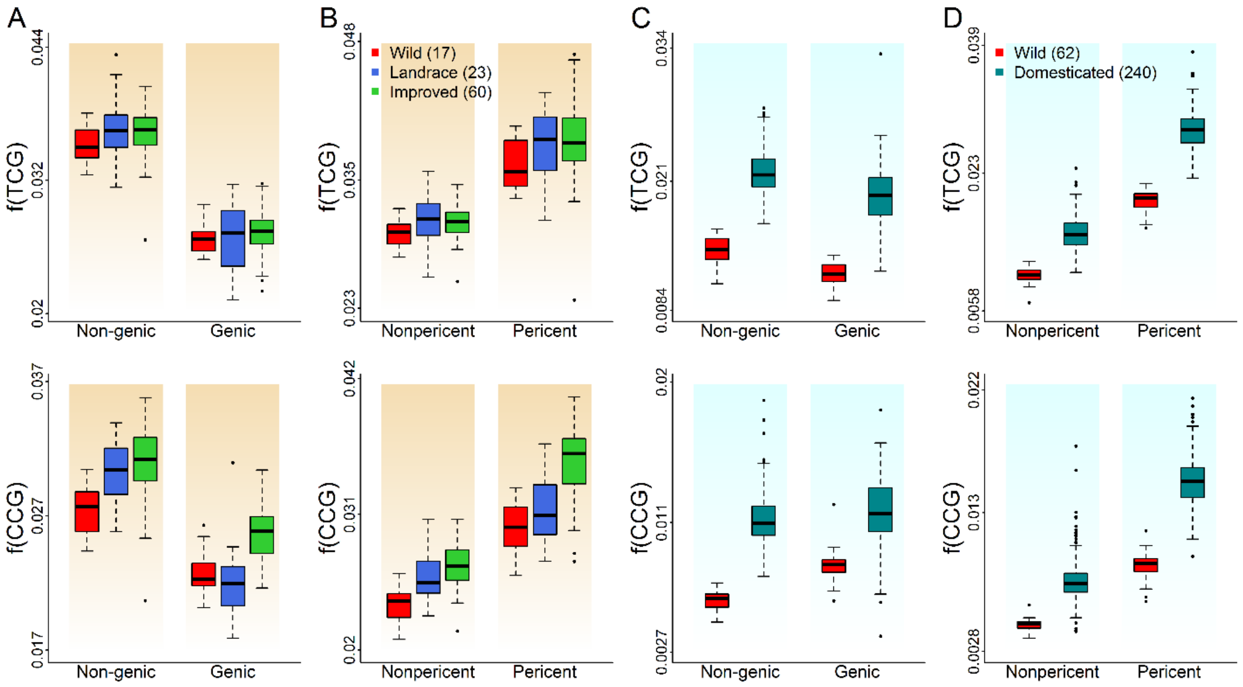

Fig. S21. Distribution of $f(\text{TCG})$ and $f(\text{CCG})$ at different genomic regions. (**A**) In maize, compare $f(\text{TCG})$ and $f(\text{CCG})$ calculated with non-genic-private SNPs to those calculated with genic-private SNPs (red for wild accession, blue for landraces, and green for improved cultivars). (**B**) In maize, compare $f(\text{TCG})$ and $f(\text{CCG})$ calculated from pericentromeric-private SNPs to those calculated from non-pericentromeric-private SNPs. (**C**) In soybean, compare $f(\text{TCG})$ and $f(\text{CCG})$ calculated with non-genic- private SNPs to those calculated with genic-private SNPs (red for wild accession, turquoise for domesticated accession). (**D**) In soybean, compare $f(\text{TCG})$ and $f(\text{CCG})$ calculated from pericentromeric-private SNPs to those calculated from non-pericentromeric-private SNPs. $f(\text{TCG})$ is the frequency of TCG→T mutation (5′-TCG-3′→5′-TTG-3′ and its reverse complement 5′-CGA-3′→5′-CAA-3′), and $f(\text{CCG})$ is the frequency of CCG→T mutation (5′-CCG-3′→5′-CTG-3′ and its reverse complement 5′-CGG-3′→5′-CAG-3′).

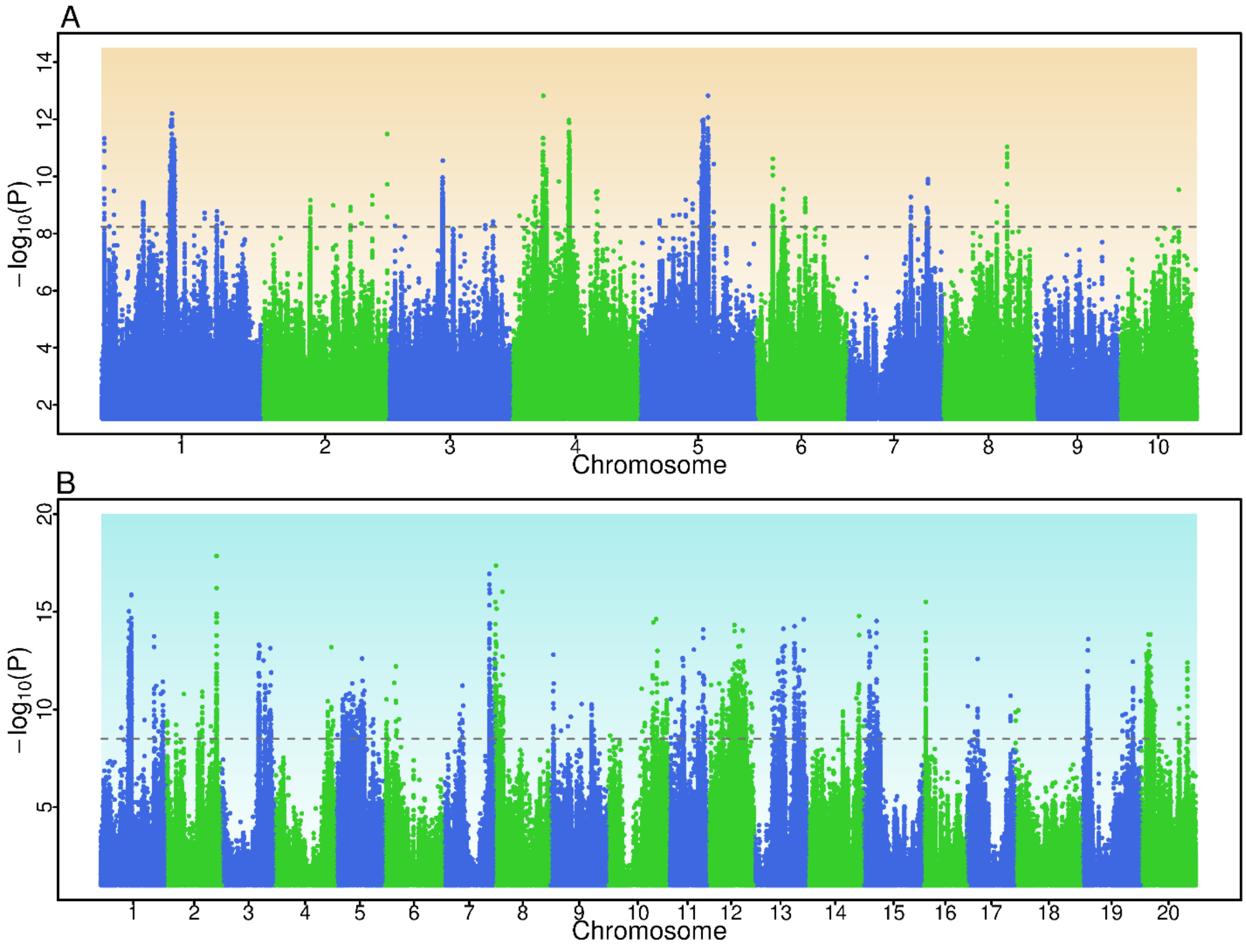

Fig. S22. GWAS-identified genomic regions underlying base-composition variation. (**A**) maize. (**B**) soybean. Manhattan plot shows the association signals detected by the mixed model between the genome-wide [AT] values across polymorphic sites in (**A**) 100 maize accessions and (**B**) 302 soybean accessions.

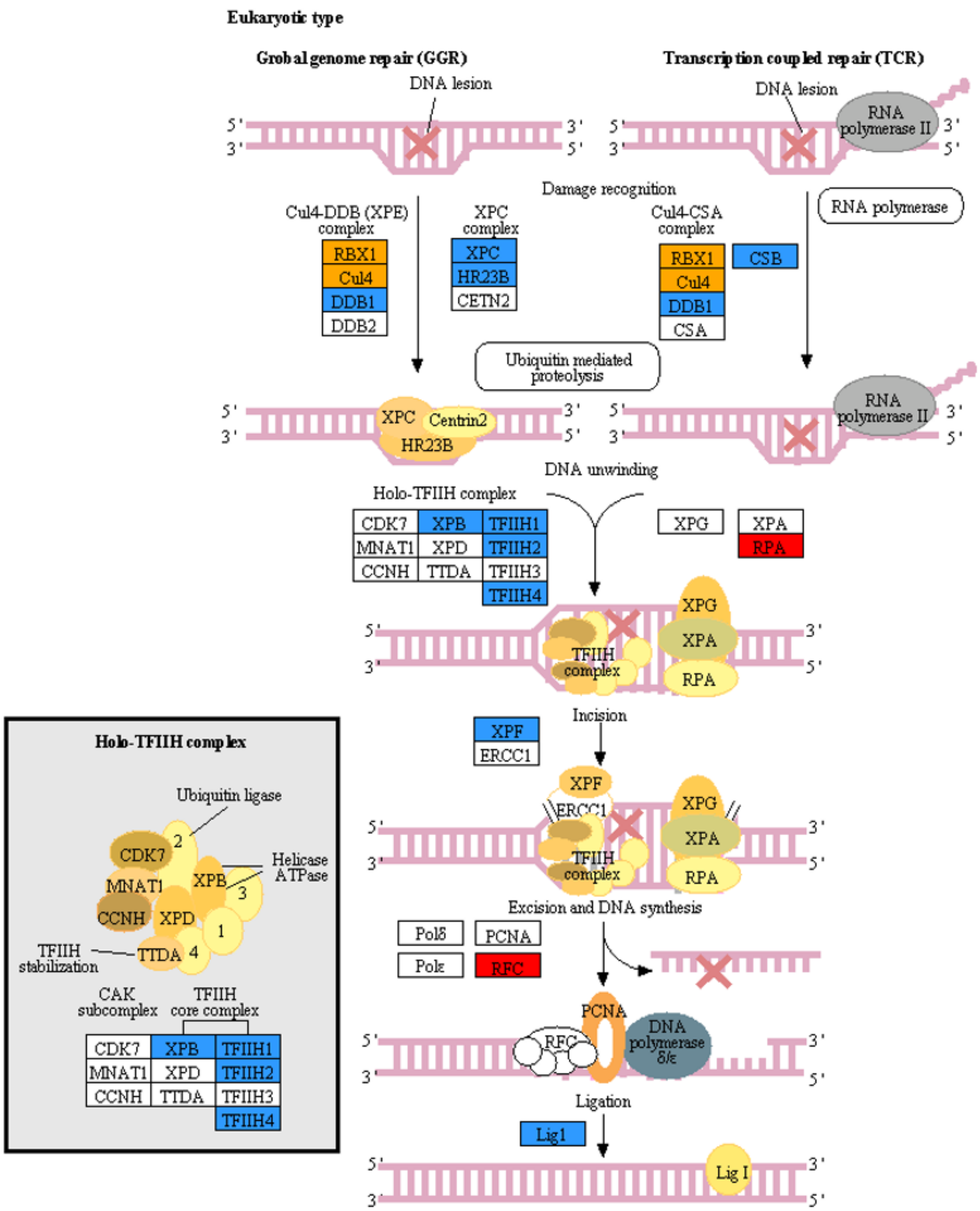

Fig. S23. GWAS tagged genes in NER pathway. The pathway was obtained from KEGG (Nucleotide excision repair, ath:03420). Genes with orange box and blue box are located within 500kb from significantly associated SNPs in maize and soybean, respectively. And genes with red box are detected in both crops.

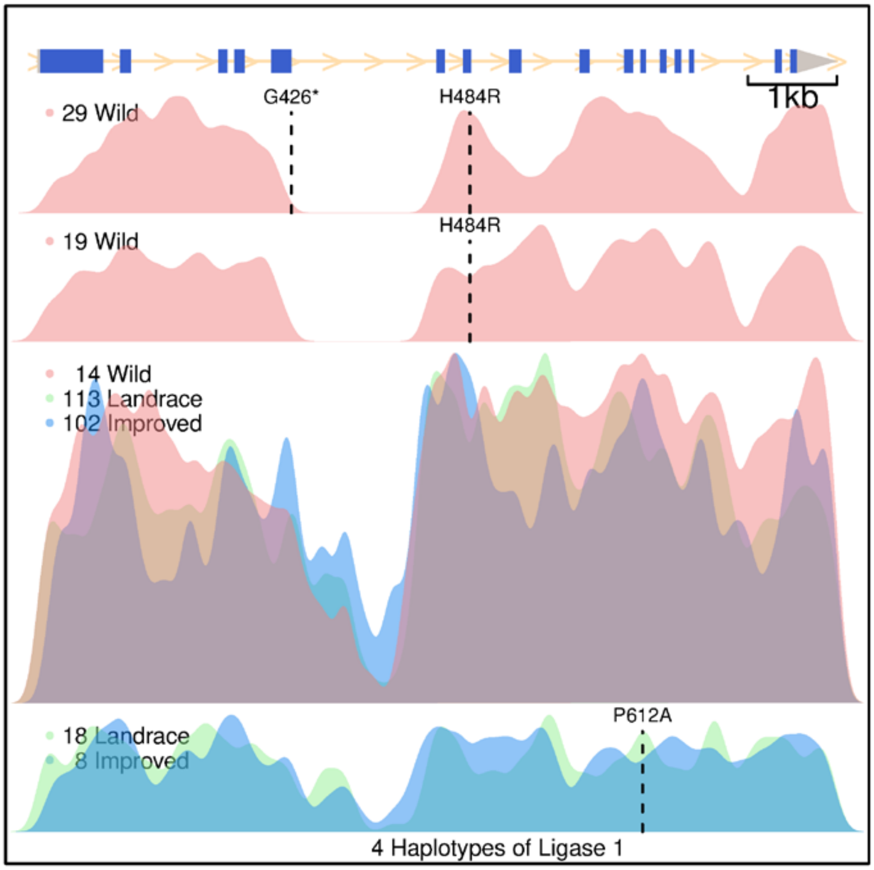

Fig. S24. Polymorphisms in soybean *DNA ligase1*. The coverage of mapped short reads was plotted to shown the indel in the 5th intron. Haplotypes of *Lig1* formed by 2 nonsynonymous SNPs and a 1.8kb indel among 302 accessions.

Supplementary Tables

Table S1. UV-related genes are enriched near the associated loci in maize.

Distance from signals (Mb)	Frequency of detected all encoded genes	Frequency of detected UV-related gene	*P*-value
0.50	1.8%	4.2%	0.002*
1.00	2.8%	5.4%	0.004*
1.50	4.0%	7.8%	0.001*
2.00	5.4%	9.6%	0.001*
2.50	6.7%	10.8%	0.002*

*, *P*-value ≤ 0.05.

Table S2. UV-related genes tagged by the associated SNPs in maize.

Genes	Chr	Start	End	*Arabidopsis* orthologue	Alias	Function
Zm00001d030376	1	127,344,674	127,353,195	AT4G19130	*RPA1*	Replication factor-A protein 1-related
Zm00001d030381	1	127,553,498	127,556,739	AT1G12370	*UVR2*	Photolyase 1
Zm00001d005361	2	171,059,897	171,064,674	AT3G53570	*CLK2B*	CDC2-related kinase subfamily
Zm00001d007897	2	241,927,996	241,932,729	AT2G02760	*ATUBC2*	UBC2 ubiquiting-conjugating enzyme
Zm00001d042988	3	185,995,285	185,995,920	AT5G01310	*APTX*	Adenylylsulfate sulfohydrolase activity, involved in base excision repair
Zm00001d049471	4	31,448,746	31,454,292	AT5G54260	*MRE11A*	DNA repair and meiotic recombination protein
Zm00001d049811	4	45,778,051	45,785,382	AT2G06510	*RPA1*	Encodes a homolog of Replication protein A
Zm00001d050085	4	64,882,472	64,888,748	AT5G46210	*CUL4*	Ubiqutin protein ligase activity, involved in DNA repair
Zm00001d050642	4	109,006,158	109,009,903	AT5G22750	*RAD5*	DNA/RNA helicase protein involves in DNA repair
Zm00001d051565	4	162,887,403	162,902,341	AT5G27740	*RFC3*	DNA repair, DNA-dependent DNA replication
Zm00001d051588	4	163,585,208	163,588,502	AT5G20570	*RBX1*	Subunit of Cul2-RING ubiquitin ligase complex, involved in nucleotide excision repair
Zm00001d014813	5	64,068,899	64,100,088	AT5G40820	*ATR*	Encodes a Arabidopsis ortholog of the ATR protein kinase
Zm00001d015871	5	127,459,293	127,465,455	AT3G48750	*CDK2*	A-type cyclin-dependent kinase, involved in DNA repair
Zm00001d021607	7	157,927,203	157,934,056	AT1G05120	*RAD16*	ATP-binding protein, required for nucleotide excision repair

Table S3. UV-related genes are enriched near the associated loci in soybean.

Distance from signals (Mb)	Frequency of detected all encoded genes	Frequency of detected UV-related gene	*P*-value
0.20	8.0%	11.2%	0.221
0.50	13.8%	20.6%	0.041*
1.00	22.1%	30.8%	0.029*
1.50	29.0%	43.0%	0.001*
2.00	33.6%	50.5%	0.001*

*, *P*-value ≤ 0.05.

Table S4. UV-related genes tagged by the associated SNPs in soybean.

Genes	Chr	Start	End	*Arabidopsis* orthologue	Alias	Function
Glyma.01g081500	1	23,600,207	23,609,665	AT3G02540	*RAD23C*	Rad23 UV excision repair protein family
Glyma.01g204500	1	53,723,913	53,729,465	AT5G41150	*UVH1*	Restriction endonuclease, type II-like superfamily protein
Glyma.01g212300	1	54,381,204	54,383,602	AT1G12370	*UVR2*	Photolyase 1
Glyma.02g182000	2	31,149,538	31,150,558	AT5G61000	*ATPRA70D*	Replication factor-A protein 1-related
Glyma.03g196800	3	40,638,356	40,640,900	AT4G18590	*AtRPA14B*	ssDNA binding protein
Glyma.04g215000	4	48,672,908	48,674,463	AT1G77470	*RFC5*	DNA-dependent ATPase required for DNA replication and repair
Glyma.04g223500	4	49,411,690	49,415,117	AT2G38560	*TFIIS*	Transcript elongation factor IIS
Glyma.08g016400	8	1,297,174	1,305,157	AT5G41370	*XPB1*	Subunit of TFIIH. 3’->5’ helicase
Glyma.08g088900	8	6,714,191	6,721,326	AT5G44740	*POLH*	Y-family DNA polymerase H
Glyma.10g156300	10	39,053,531	39,057,116	AT3G05210	*UVR7*	Nucleotide repair protein
Glyma.10g193000	10	42,527,991	42,540,681	AT1G55750	*AtTFB1-1*	Core TFIIH subunits
Glyma.11g038500	11	2,750,496	2,758,704	AT5G41150	*UVH1*	Restriction endonuclease, type II-like superfamily protein
Glyma.11g193100	11	26,629,471	26,638,425	AT1G08130	*LIG1*	DNA ligase 1
Glyma.11g211200	11	30,385,621	30,386,438	AT4G17020	*AtTFB2*	Core TFIIH subunits
Glyma.12g096100	12	8,101,497	8,111,369	AT5G22010	*RFC1*	Replication factor C1
Glyma.12g106200	12	9,701,540	9,702,031	AT4G21100	*DDB1B*	Damaged DNA binding protein 1B
Glyma.13g096800	13	21,172,838	21,179,791	AT3G02920	*RPA32B*	Replication protein A, subunit RPA32
Glyma.13g245700	13	35,464,732	35,471,257	AT5G28740		TPR-like superfamily protein
Glyma.15g055100	15	4,326,815	4,329,488	AT3G50360	*CEN2*	Centrin2
Glyma.15g068100	15	5,200,760	5,206,464	AT5G28740		TPR-like superfamily protein
Glyma.19g044000	19	6,474,307	6,487,275	AT1G16710	*HAC12*	Histone acetyltransferase of the CBP family 12
Glyma.19g129000	19	38,832,877	38,838,251	AT1G05055	*GTF2H2*	General transcription factor II H2

Table S5. Summary of 100 maize accessions. (Nature Genetics 2012, 44:803–807).

Accession	Category	Species	Class
TIL01	Parviglumis	*Z. mays* ssp. *parviglumis*	TIL
TIL03	Parviglumis	*Z. mays* ssp. *parviglumis*	TIL
TIL04 (TIP-454)	Parviglumis	*Z. mays* ssp. *parviglumis*	TIL
TIL05	Parviglumis	*Z. mays* ssp. *parviglumis*	TIL
TIL06 (TIP-260)	Parviglumis	*Z. mays* ssp. *parviglumis*	TIL
TIL06 (TIP-496)	Parviglumis	*Z. mays* ssp. *parviglumis*	TIL
TIL07	Parviglumis	*Z. mays* ssp. *parviglumis*	TIL
TIL08	Mexicana	*Z. mays* ssp. *mexicana*	TIL
TIL09	Parviglumis	*Z. mays* ssp. *parviglumis*	TIL
TIL10	Parviglumis	*Z. mays* ssp. *parviglumis*	TIL
TIL11	Parviglumis	*Z. mays* ssp. *parviglumis*	TIL
TIL12	Parviglumis	*Z. mays* ssp. *parviglumis*	TIL
TIL14	Parviglumis	*Z. mays* ssp. *parviglumis*	TIL
TIL15	Parviglumis	*Z. mays* ssp. *parviglumis*	TIL
TIL16	Parviglumis	*Z. mays* ssp. *parviglumis*	TIL
TIL17	Parviglumis	*Z. mays* ssp. *parviglumis*	TIL
TIL25	Mexicana	*Z. mays* ssp. *mexicana*	TIL
MR01	Landrace	*Z. mays* ssp. *mays*	LRI
MR02	Landrace	*Z. mays* ssp. *mays*	LRI
MR03	Landrace	*Z. mays* ssp. *mays*	LRI
MR05	Landrace	*Z. mays* ssp. *mays*	LRI
MR06	Landrace	*Z. mays* ssp. *mays*	LRI
MR07	Landrace	*Z. mays* ssp. *mays*	LRI
MR08	Landrace	*Z. mays* ssp. *mays*	LRI
MR09	Landrace	*Z. mays* ssp. *mays*	LRI
MR10	Landrace	*Z. mays* ssp. *mays*	LRI
MR11	Landrace	*Z. mays* ssp. *mays*	LRI
MR12	Landrace	*Z. mays* ssp. *mays*	LRI
MR13	Landrace	*Z. mays* ssp. *mays*	LRI
MR14	Landrace	*Z. mays* ssp. *mays*	LRI
MR17	Landrace	*Z. mays* ssp. *mays*	LRI
MR18	Landrace	*Z. mays* ssp. *mays*	LRI
MR19	Landrace	*Z. mays* ssp. *mays*	LRI
MR20	Landrace	*Z. mays* ssp. *mays*	LRI
MR21	Landrace	*Z. mays* ssp. *mays*	LRI
MR22	Landrace	*Z. mays* ssp. *mays*	LRI
MR23	Landrace	*Z. mays* ssp. *mays*	LRI
MR24	Landrace	*Z. mays* ssp. *mays*	LRI
MR25	Landrace	*Z. mays* ssp. *mays*	LRI
MR26	Landrace	*Z. mays* ssp. *mays*	LRI
B73	Improved	*Z. mays* ssp. *mays*	SS
B97	Improved	*Z. mays* ssp. *mays*	NSS
CAU178	Improved	*Z. mays* ssp. *mays*	CAU
CAU478	Improved	*Z. mays* ssp. *mays*	CAU
CAU5003	Improved	*Z. mays* ssp. *mays*	CAU
CAUCHANG72	Improved	*Z. mays* ssp. *mays*	CAU
CAUMO17	Improved	*Z. mays* ssp. *mays*	NSS
CAUZHENG58	Improved	*Z. mays* ssp. *mays*	CAU
CML103	Improved	*Z. mays* ssp. *mays*	TS
CML133	Improved	*Z. mays* ssp. *mays*	CML
CML192	Improved	*Z. mays* ssp. *mays*	CML

CML202	Improved	*Z. mays* ssp. *mays*	CML
CML206	Improved	*Z. mays* ssp. *mays*	CML
CML228	Improved	*Z. mays* ssp. *mays*	TS
CML247	Improved	*Z. mays* ssp. *mays*	TS
CML277	Improved	*Z. mays* ssp. *mays*	TS
CML312SR	Improved	*Z. mays* ssp. *mays*	CML
CML322	Improved	*Z. mays* ssp. *mays*	TS
CML330	Improved	*Z. mays* ssp. *mays*	CML
CML333	Improved	*Z. mays* ssp. *mays*	TS
CML341	Improved	*Z. mays* ssp. *mays*	TS
CML411	Improved	*Z. mays* ssp. *mays*	CML
CML418	Improved	*Z. mays* ssp. *mays*	CML
CML479	Improved	*Z. mays* ssp. *mays*	CML
CML504	Improved	*Z. mays* ssp. *mays*	CML
CML505	Improved	*Z. mays* ssp. *mays*	CML
CML511	Improved	*Z. mays* ssp. *mays*	CML
CML52	Improved	*Z. mays* ssp. *mays*	TS
CML69	Improved	*Z. mays* ssp. *mays*	TS
CML84	Improved	*Z. mays* ssp. *mays*	CML
CML85	Improved	*Z. mays* ssp. *mays*	CML
CML96	Improved	*Z. mays* ssp. *mays*	CML
CML99	Improved	*Z. mays* ssp. *mays*	CML
H16	Improved	*Z. mays* ssp. *mays*	Not Assigned
HP301	Improved	*Z. mays* ssp. *mays*	POPCORN
IL14H	Improved	*Z. mays* ssp. *mays*	SWEET
KI11	Improved	*Z. mays* ssp. *mays*	TS
KI3	Improved	*Z. mays* ssp. *mays*	TS
KY21	Improved	*Z. mays* ssp. *mays*	NSS
M162W	Improved	*Z. mays* ssp. *mays*	NSS
M37W	Improved	*Z. mays* ssp. *mays*	MIXED
MO17	Improved	*Z. mays* ssp. *mays*	NSS
MO18W	Improved	*Z. mays* ssp. *mays*	MIXED
MS71	Improved	*Z. mays* ssp. *mays*	NSS
NC350	Improved	*Z. mays* ssp. *mays*	TS
NC358	Improved	*Z. mays* ssp. *mays*	TS
OH43	Improved	*Z. mays* ssp. *mays*	NSS
OH7B	Improved	*Z. mays* ssp. *mays*	NSS
P1	Improved	*Z. mays* ssp. *mays*	Not Assigned
P39	Improved	*Z. mays* ssp. *mays*	SWEET
TX303	Improved	*Z. mays* ssp. *mays*	MIXED
TZI8	Improved	*Z. mays* ssp. *mays*	TS
VL0512447	Improved	*Z. mays* ssp. *mays*	Chinese Tropical
VL05128	Improved	*Z. mays* ssp. *mays*	Chinese Tropical
VL054178	Improved	*Z. mays* ssp. *mays*	Chinese Tropical
VL05610	Improved	*Z. mays* ssp. *mays*	Chinese Tropical
VL056883	Improved	*Z. mays* ssp. *mays*	Chinese Tropical
VL062784	Improved	*Z. mays* ssp. *mays*	Chinese Tropical
W22	Improved	*Z. mays* ssp. *mays*	NSS
W64A	Improved	*Z. mays* ssp. *mays*	NSS

Table S6. Summary of 302 soybean accessions. (Nature Biotechnology 2015, 33:408–414).

Accession	Category	Species	PI CGN# & Name
IGDB-001	G. soja	*G. soja* Siebold & Zucc.	ZJ-ZY020
IGDB-002	G. soja	*G. soja* Siebold & Zucc.	ZJ-YJ086
IGDB-003	G. soja	*G. soja* Siebold & Zucc.	ZJ-Y314
IGDB-004	G. soja	*G. soja* Siebold & Zucc.	ZJ-Y217
IGDB-005	G. soja	*G. soja* Siebold & Zucc.	ZJ-Y200
IGDB-006	G. soja	*G. soja* Siebold & Zucc.	ZJ-Y191
IGDB-007	G. soja	*G. soja* Siebold & Zucc.	ZJ-Y188
IGDB-008	G. soja	*G. soja* Siebold & Zucc.	ZJ-Y108
IGDB-009	G. soja	*G. soja* Siebold & Zucc.	ZJ-YJ038
IGDB-010	G. soja	*G. soja* Siebold & Zucc.	ZJ-Y282
IGDB-011	G. soja	*G. soja* Siebold & Zucc.	ZJ-Y2300-1
IGDB-012	G. soja	*G. soja* Siebold & Zucc.	ZJ-Y155
IGDB-013	G. soja	*G. soja* Siebold & Zucc.	PI 597461C
IGDB-014	G. soja	*G. soja* Siebold & Zucc.	PI 597461A
IGDB-015	G. soja	*G. soja* Siebold & Zucc.	PI 597459D
IGDB-016	G. soja	*G. soja* Siebold & Zucc.	PI 597459C
IGDB-017	G. soja	*G. soja* Siebold & Zucc.	PI 593983
IGDB-018	G. soja	*G. soja* Siebold & Zucc.	PI 578357
IGDB-019	G. soja	*G. soja* Siebold & Zucc.	PI 578341
IGDB-020	G. soja	*G. soja* Siebold & Zucc.	PI 562565
IGDB-021	G. soja	*G. soja* Siebold & Zucc.	PI 562559
IGDB-022	G. soja	*G. soja* Siebold & Zucc.	PI 549046
IGDB-023	G. soja	*G. soja* Siebold & Zucc.	PI 547831
IGDB-024	G. soja	*G. soja* Siebold & Zucc.	PI 522228
IGDB-025	G. soja	*G. soja* Siebold & Zucc.	PI 522226
IGDB-026	G. soja	*G. soja* Siebold & Zucc.	PI 522216
IGDB-027	G. soja	*G. soja* Siebold & Zucc.	PI 522182B
IGDB-028	G. soja	*G. soja* Siebold & Zucc.	PI 507662
IGDB-029	G. soja	*G. soja* Siebold & Zucc.	PI 504286
IGDB-030	G. soja	*G. soja* Siebold & Zucc.	PI 483465
IGDB-031	G. soja	*G. soja* Siebold & Zucc.	PI 483464A
IGDB-032	G. soja	*G. soja* Siebold & Zucc.	PI 483460B
IGDB-033	G. soja	*G. soja* Siebold & Zucc.	PI 479769
IGDB-034	G. soja	*G. soja* Siebold & Zucc.	PI 479752
IGDB-035	G. soja	*G. soja* Siebold & Zucc.	PI 468916
IGDB-036	G. soja	*G. soja* Siebold & Zucc.	PI 468400A
IGDB-037	G. soja	*G. soja* Siebold & Zucc.	PI 464935
IGDB-038	G. soja	*G. soja* Siebold & Zucc.	PI 464929B
IGDB-039	G. soja	*G. soja* Siebold & Zucc.	PI 464929A
IGDB-040	G. soja	*G. soja* Siebold & Zucc.	PI 464927A
IGDB-041	G. soja	*G. soja* Siebold & Zucc.	PI 458538
IGDB-042	G. soja	*G. soja* Siebold & Zucc.	PI 458536
IGDB-043	G. soja	*G. soja* Siebold & Zucc.	PI 458535
IGDB-044	G. soja	*G. soja* Siebold & Zucc.	PI 447004
IGDB-045	G. soja	*G. soja* Siebold & Zucc.	PI 424096
IGDB-046	G. soja	*G. soja* Siebold & Zucc.	PI 423991
IGDB-047	G. soja	*G. soja* Siebold & Zucc.	PI 407301
IGDB-048	G. soja	*G. soja* Siebold & Zucc.	PI 407288
IGDB-049	G. soja	*G. soja* Siebold & Zucc.	PI 407285
IGDB-050	G. soja	*G. soja* Siebold & Zucc.	PI 407275
IGDB-051	G. soja	*G. soja* Siebold & Zucc.	PI 407246

IGDB-052	G. soja	*G. soja* Siebold & Zucc.	PI 407197
IGDB-053	G. soja	*G. soja* Siebold & Zucc.	PI 407170
IGDB-054	G. soja	*G. soja* Siebold & Zucc.	PI 407131
IGDB-055	G. soja	*G. soja* Siebold & Zucc.	PI 407027
IGDB-056	G. soja	*G. soja* Siebold & Zucc.	PI 393551
IGDB-057	G. soja	*G. soja* Siebold & Zucc.	PI 378692
IGDB-058	G. soja	*G. soja* Siebold & Zucc.	PI 366123
IGDB-059	G. soja	*G. soja* Siebold & Zucc.	PI 366121
IGDB-060	G. soja	*G. soja* Siebold & Zucc.	PI 366120
IGDB-061	G. soja	*G. soja* Siebold & Zucc.	PI 339871A
IGDB-062	G. soja	*G. soja* Siebold & Zucc.	PI 326582A
IGDB-063	Landrace	*G. max* (L.) Merr.	Yu Shi Dou
IGDB-064	Landrace	*G. max* (L.) Merr.	Yu Jiang Wu Yue Niu Mao Huang
IGDB-065	Landrace	*G. max* (L.) Merr.	You Pi Zhi Hei Dou
IGDB-066	Landrace	*G. max* (L.) Merr.	Yi Zheng Da Li Huang Dou
IGDB-067	Landrace	*G. max* (L.) Merr.	Xin Xian Xiao Huang Dou
IGDB-068	Landrace	*G. max* (L.) Merr.	Xiao Mi Dou
IGDB-069	Landrace	*G. max* (L.) Merr.	Xiao Huang Dou
IGDB-070*	Landrace	*G. max* (L.) Merr.	Xiao Bai Qi
IGDB-071	Landrace	*G. max* (L.) Merr.	Xiao Bai Dou
IGDB-072	Landrace	*G. max* (L.) Merr.	Xiang Dou No.4
IGDB-073	Landrace	*G. max* (L.) Merr.	Xia Men Teng Zai Dou
IGDB-074	Landrace	*G. max* (L.) Merr.	Xia Hei Dou
IGDB-075	Landrace	*G. max* (L.) Merr.	Tong an Zi Hong Dou
IGDB-076	Landrace	*G. max* (L.) Merr.	Tian E Dan
IGDB-077	Landrace	*G. max* (L.) Merr.	Tai Xin Niu Mao Huang Yi
IGDB-078	Landrace	*G. max* (L.) Merr.	Sha Xin Dou
IGDB-079	Landrace	*G. max* (L.) Merr.	Sha Xian Wu Dou
IGDB-080	Landrace	*G. max* (L.) Merr.	Sha Xian Qin Dou
IGDB-081	Landrace	*G. max* (L.) Merr.	Qing Dou
IGDB-082	Landrace	*G. max* (L.) Merr.	PI Xian Nian Zhuang Liu Yue Xian
IGDB-083	Landrace	*G. max* (L.) Merr.	PI Xian Da Zi Huo Cao
IGDB-084	Landrace	*G. max* (L.) Merr.	PI 89138
IGDB-085	Landrace	*G. max* (L.) Merr.	PI 88479
IGDB-086	Landrace	*G. max* (L.) Merr.	PI 86024
IGDB-087	Landrace	*G. max* (L.) Merr.	PI 84987A
IGDB-088	Landrace	*G. max* (L.) Merr.	PI 84987
IGDB-089	Landrace	*G. max* (L.) Merr.	PI 84631
IGDB-090	Landrace	*G. max* (L.) Merr.	PI 83945-3
IGDB-091	Landrace	*G. max* (L.) Merr.	PI 80837
IGDB-092	Landrace	*G. max* (L.) Merr.	PI 80822
IGDB-093	Improved	*G. max* (L.) Merr.	PI 634883
IGDB-094	Landrace	*G. max* (L.) Merr.	PI 603756
IGDB-095	Landrace	*G. max* (L.) Merr.	PI 603675
IGDB-096	Landrace	*G. max* (L.) Merr.	PI 603596
IGDB-097	Landrace	*G. max* (L.) Merr.	PI 603516
IGDB-098	Landrace	*G. max* (L.) Merr.	PI 603424A
IGDB-099	Landrace	*G. max* (L.) Merr.	PI 603420
IGDB-100*	Landrace	*G. max* (L.) Merr.	PI 603384
IGDB-101	Landrace	*G. max* (L.) Merr.	PI 603357
IGDB-102	Landrace	*G. max* (L.) Merr.	PI 603336
IGDB-103	Landrace	*G. max* (L.) Merr.	PI 603318
IGDB-104	Landrace	*G. max* (L.) Merr.	PI 602991
IGDB-105	Landrace	*G. max* (L.) Merr.	PI 594788

IGDB-106	Landrace	*G. max* (L.) Merr.	PI 594777
IGDB-107	Landrace	*G. max* (L.) Merr.	PI 594629
IGDB-108	Landrace	*G. max* (L.) Merr.	PI 594615
IGDB-109	Landrace	*G. max* (L.) Merr.	PI 594579
IGDB-110	Landrace	*G. max* (L.) Merr.	PI 594451
IGDB-111	Landrace	*G. max* (L.) Merr.	PI 594301
IGDB-112	Improved	*G. max* (L.) Merr.	PI 591511
IGDB-113	Improved	*G. max* (L.) Merr.	PI 591495
IGDB-114	Landrace	*G. max* (L.) Merr.	PI 588053A
IGDB-115	Landrace	*G. max* (L.) Merr.	PI 587848
IGDB-116	Landrace	*G. max* (L.) Merr.	PI 587752
IGDB-117	Landrace	*G. max* (L.) Merr.	PI 587666
IGDB-118	Landrace	*G. max* (L.) Merr.	PI 587552
IGDB-119	Landrace	*G. max* (L.) Merr.	PI 578457A
IGDB-120	Landrace	*G. max* (L.) Merr.	PI 567525
IGDB-121	Landrace	*G. max* (L.) Merr.	PI 567503
IGDB-122	Landrace	*G. max* (L.) Merr.	PI 567395
IGDB-123	Landrace	*G. max* (L.) Merr.	PI 567364
IGDB-124	Landrace	*G. max* (L.) Merr.	PI 567298
IGDB-125	Landrace	*G. max* (L.) Merr.	PI 567293
IGDB-126	Landrace	*G. max* (L.) Merr.	PI 567258
IGDB-127	Landrace	*G. max* (L.) Merr.	PI 567189A
IGDB-128	Landrace	*G. max* (L.) Merr.	PI 567071A
IGDB-129	Landrace	*G. max* (L.) Merr.	PI 548488
IGDB-130	Landrace	*G. max* (L.) Merr.	PI 548485
IGDB-131	Improved	*G. max* (L.) Merr.	PI 548477
IGDB-132	Landrace	*G. max* (L.) Merr.	PI 548456
IGDB-133	Landrace	*G. max* (L.) Merr.	PI 548445
IGDB-134	Landrace	*G. max* (L.) Merr.	PI 548417
IGDB-135	Landrace	*G. max* (L.) Merr.	PI 548406
IGDB-136	Landrace	*G. max* (L.) Merr.	PI 548402
IGDB-137	Landrace	*G. max* (L.) Merr.	PI 548391
IGDB-138	Landrace	*G. max* (L.) Merr.	PI 548382
IGDB-139	Landrace	*G. max* (L.) Merr.	PI 548379
IGDB-140	Improved	*G. max* (L.) Merr.	PI 548362
IGDB-141	Landrace	*G. max* (L.) Merr.	PI 548348
IGDB-142	Landrace	*G. max* (L.) Merr.	PI 548342
IGDB-143	Improved	*G. max* (L.) Merr.	PI 548311
IGDB-144	Landrace	*G. max* (L.) Merr.	PI 548298
IGDB-145	Improved	*G. max* (L.) Merr.	PI 548190
IGDB-146	Improved	*G. max* (L.) Merr.	PI 548182
IGDB-147	Improved	*G. max* (L.) Merr.	PI 547562
IGDB-148	Landrace	*G. max* (L.) Merr.	PI 507355
IGDB-149	Landrace	*G. max* (L.) Merr.	PI 467343
IGDB-150	Landrace	*G. max* (L.) Merr.	PI 438498
IGDB-151	Landrace	*G. max* (L.) Merr.	PI 437944
IGDB-152	Landrace	*G. max* (L.) Merr.	PI 437679
IGDB-153	Landrace	*G. max* (L.) Merr.	PI 437654
IGDB-154	Landrace	*G. max* (L.) Merr.	PI 437653
IGDB-155	Landrace	*G. max* (L.) Merr.	PI 437321
IGDB-156	Landrace	*G. max* (L.) Merr.	PI 424391
IGDB-157	Landrace	*G. max* (L.) Merr.	PI 423967
IGDB-158	Landrace	*G. max* (L.) Merr.	PI 423954
IGDB-159	Landrace	*G. max* (L.) Merr.	PI 417398

IGDB-160	Landrace	*G. max* (L.) Merr.	PI 416971
IGDB-161	Landrace	*G. max* (L.) Merr.	PI 416890
IGDB-162	Landrace	*G. max* (L.) Merr.	PI 407849
IGDB-163	Landrace	*G. max* (L.) Merr.	PI 407801
IGDB-164	Landrace	*G. max* (L.) Merr.	PI 407716
IGDB-165	Landrace	*G. max* (L.) Merr.	PI 404182
IGDB-166	Landrace	*G. max* (L.) Merr.	PI 399043
IGDB-167	Landrace	*G. max* (L.) Merr.	PI 398296
IGDB-168	Landrace	*G. max* (L.) Merr.	PI 339734
IGDB-169	Landrace	*G. max* (L.) Merr.	PI 323576
IGDB-170	Landrace	*G. max* (L.) Merr.	PI 317336
IGDB-171	Landrace	*G. max* (L.) Merr.	PI 317334A
IGDB-172	Landrace	*G. max* (L.) Merr.	PI 253658B
IGDB-173	Landrace	*G. max* (L.) Merr.	PI 243541
IGDB-174	Landrace	*G. max* (L.) Merr.	PI 196166
IGDB-175	Landrace	*G. max* (L.) Merr.	PI 157421
IGDB-176	Landrace	*G. max* (L.) Merr.	PI 153262
IGDB-177	Landrace	*G. max* (L.) Merr.	Pei Xian Xiao You Dou
IGDB-178	Landrace	*G. max* (L.) Merr.	Nian Shi Huang Dou
IGDB-179	Landrace	*G. max* (L.) Merr.	Ni Dou
IGDB-180	Landrace	*G. max* (L.) Merr.	Ni Ding Hua Mei Dou
IGDB-181*	Landrace	*G. max* (L.) Merr.	Nan Guan Xiao PI Qing
IGDB-182	Landrace	*G. max* (L.) Merr.	Long quan Da Dou
IGDB-183	Landrace	*G. max* (L.) Merr.	Jin Shan Cha Zhu Shi Dou
IGDB-184	Landrace	*G. max* (L.) Merr.	Jin Huang No.35
IGDB-185	Landrace	*G. max* (L.) Merr.	Ji Shan De Da Li Hei Dou
IGDB-186*	Landrace	*G. max* (L.) Merr.	Hu Pi Dou
IGDB-187	Landrace	*G. max* (L.) Merr.	Hong Zhu Dou
IGDB-188	Landrace	*G. max* (L.) Merr.	Hong Hu Liu Yue Bao
IGDB-189	Landrace	*G. max* (L.) Merr.	Hei Wa Shi Dou
IGDB-190	Landrace	*G. max* (L.) Merr.	Hei He Xiao Huang Dou
IGDB-191	Landrace	*G. max* (L.) Merr.	He Dou
IGDB-192	Landrace	*G. max* (L.) Merr.	Guang Rao Da Qing Dou
IGDB-193	Improved	*G. max* (L.) Merr.	FC 33243
IGDB-194	Landrace	*G. max* (L.) Merr.	Dong Shan Bai Ma Dou
IGDB-195	Landrace	*G. max* (L.) Merr.	Dai Mi Dou
IGDB-196	Landrace	*G. max* (L.) Merr.	Da Tun Xiao Hei Dou
IGDB-197	Landrace	*G. max* (L.) Merr.	Da Qing Ren
IGDB-198	Landrace	*G. max* (L.) Merr.	Da Li Huang
IGDB-199	Landrace	*G. max* (L.) Merr.	Cu Dou
IGDB-200	Landrace	*G. max* (L.) Merr.	Bin Hai Da huang Ke Zi Jia
IGDB-201	Improved	*G. max* (L.) Merr.	Beijing-IGDB-1
IGDB-202	Landrace	*G. max* (L.) Merr.	Bai Mao Dou
IGDB-203	Landrace	*G. max* (L.) Merr.	Bai Lu Dou
IGDB-204	Improved	*G. max* (L.) Merr.	Zhong Huang No.50
IGDB-205	Improved	*G. max* (L.) Merr.	Zhong Huang No.40
IGDB-206	Improved	*G. max* (L.) Merr.	Zhong Huang No.38
IGDB-207	Improved	*G. max* (L.) Merr.	Zhong Huang No.35
IGDB-208	Improved	*G. max* (L.) Merr.	Zhong Huang No.31
IGDB-209	Improved	*G. max* (L.) Merr.	Zhong Huang No.14
IGDB-210	Improved	*G. max* (L.) Merr.	Zhong Huang No.13
IGDB-211	Improved	*G. max* (L.) Merr.	Xi Zang Da Dou No.20
IGDB-212	Improved	*G. max* (L.) Merr.	Wei No.6823
IGDB-213	Improved	*G. max* (L.) Merr.	Tie Feng No.22

IGDB-214	Improved	*G. max* (L.) Merr.	Tai Wan No.1
IGDB-215	Improved	*G. max* (L.) Merr.	Su Nong No.33
IGDB-216	Improved	*G. max* (L.) Merr.	Su Nong No.25
IGDB-217	Improved	*G. max* (L.) Merr.	Su Nong No.14
IGDB-218	Improved	*G. max* (L.) Merr.	Su Nong No.10
IGDB-219	Improved	*G. max* (L.) Merr.	Shu Xian No.205
IGDB-220	Improved	*G. max* (L.) Merr.	Sheng Dou No.9
IGDB-221	Improved	*G. max* (L.) Merr.	Shen Li No.3
IGDB-222	Improved	*G. max* (L.) Merr.	Harbin 91-6065
IGDB-223	Improved	*G. max* (L.) Merr.	PI 591541
IGDB-224	Improved	*G. max* (L.) Merr.	PI 591435
IGDB-225	Improved	*G. max* (L.) Merr.	PI 591433
IGDB-226	Improved	*G. max* (L.) Merr.	PI 591432
IGDB-227	Improved	*G. max* (L.) Merr.	PI 591431
IGDB-228	Improved	*G. max* (L.) Merr.	PI 553047
IGDB-229	Improved	*G. max* (L.) Merr.	PI 548985
IGDB-230	Improved	*G. max* (L.) Merr.	PI 548657
IGDB-231	Improved	*G. max* (L.) Merr.	PI 548644
IGDB-232	Improved	*G. max* (L.) Merr.	PI 548643
IGDB-233	Improved	*G. max* (L.) Merr.	PI 548638
IGDB-234	Improved	*G. max* (L.) Merr.	PI 548634
IGDB-235	Improved	*G. max* (L.) Merr.	PI 548631
IGDB-236	Improved	*G. max* (L.) Merr.	PI 548604
IGDB-237	Improved	*G. max* (L.) Merr.	PI 548603
IGDB-238	Improved	*G. max* (L.) Merr.	PI 548593
IGDB-239	Improved	*G. max* (L.) Merr.	PI 548573
IGDB-240	Improved	*G. max* (L.) Merr.	PI 548565
IGDB-241	Improved	*G. max* (L.) Merr.	PI 548540
IGDB-242	Improved	*G. max* (L.) Merr.	PI 548524
IGDB-243	Improved	*G. max* (L.) Merr.	PI 548520
IGDB-244	Improved	*G. max* (L.) Merr.	PI 548512
IGDB-245	Improved	*G. max* (L.) Merr.	PI 547779
IGDB-246	Improved	*G. max* (L.) Merr.	PI 547716
IGDB-247	Improved	*G. max* (L.) Merr.	PI 547690
IGDB-248	Improved	*G. max* (L.) Merr.	PI 547686
IGDB-249	Improved	*G. max* (L.) Merr.	PI 547680
IGDB-250	Improved	*G. max* (L.) Merr.	PI 547488
IGDB-251	Improved	*G. max* (L.) Merr.	PI 547460
IGDB-252	Improved	*G. max* (L.) Merr.	PI 547459
IGDB-253	Improved	*G. max* (L.) Merr.	PI 547409
IGDB-254	Improved	*G. max* (L.) Merr.	PI 546044
IGDB-255	Improved	*G. max* (L.) Merr.	PI 542403
IGDB-256	Improved	*G. max* (L.) Merr.	PI 540552
IGDB-257	Improved	*G. max* (L.) Merr.	PI 536635
IGDB-258	Improved	*G. max* (L.) Merr.	PI 533655
IGDB-259	Improved	*G. max* (L.) Merr.	PI 533602
IGDB-260	Improved	*G. max* (L.) Merr.	PI 518750
IGDB-261	Improved	*G. max* (L.) Merr.	Jilin 21
IGDB-262	Improved	*G. max* (L.) Merr.	PI 518664
IGDB-263	Improved	*G. max* (L.) Merr.	PI 515961
IGDB-264	Improved	*G. max* (L.) Merr.	PI 513382
IGDB-265	Improved	*G. max* (L.) Merr.	PI 508266
IGDB-266	Improved	*G. max* (L.) Merr.	PI 508083

IGDB-267	Improved	*G. max* (L.) Merr.	Nan Nong Cai Dou No.1
IGDB-268	Improved	*G. max* (L.) Merr.	Lu Dou No.11
IGDB-269	Improved	*G. max* (L.) Merr.	Liao Dou No.3
IGDB-270	Improved	*G. max* (L.) Merr.	Liao Dou No.21
IGDB-271	Improved	*G. max* (L.) Merr.	Liao Dou No.17
IGDB-272	Improved	*G. max* (L.) Merr.	Liao Dou No.15
IGDB-273	Improved	*G. max* (L.) Merr.	Liao Dou No.11
IGDB-274	Improved	*G. max* (L.) Merr.	Jiu Nong No.30
IGDB-275	Improved	*G. max* (L.) Merr.	Jin Da No.75
IGDB-276	Improved	*G. max* (L.) Merr.	Jin Da No.73
IGDB-277	Improved	*G. max* (L.) Merr.	Jin Da No.70
IGDB-278	Improved	*G. max* (L.) Merr.	Jin Da No.62
IGDB-279	Improved	*G. max* (L.) Merr.	Jin Da No.52
IGDB-280	Improved	*G. max* (L.) Merr.	Jin Da No.26
IGDB-281	Improved	*G. max* (L.) Merr.	Ji Yu No.90
IGDB-282	Improved	*G. max* (L.) Merr.	Hei Nong No.51
IGDB-283	Improved	*G. max* (L.) Merr.	Hei He No.1
IGDB-284	Improved	*G. max* (L.) Merr.	He Feng No.25
IGDB-285	Improved	*G. max* (L.) Merr.	He Feng No.23
IGDB-286	Improved	*G. max* (L.) Merr.	He Dou No.13
IGDB-287	Improved	*G. max* (L.) Merr.	Gui Chun No.8
IGDB-288	Improved	*G. max* (L.) Merr.	Fen Dou No.89
IGDB-289	Improved	*G. max* (L.) Merr.	Fen Dou No.88
IGDB-290	Improved	*G. max* (L.) Merr.	Fen Dou No.86
IGDB-291	Improved	*G. max* (L.) Merr.	Fen Dou No.85
IGDB-292	Improved	*G. max* (L.) Merr.	Fen Dou No.79
IGDB-293	Improved	*G. max* (L.) Merr.	Fen Dou No.78
IGDB-294	Improved	*G. max* (L.) Merr.	Fen Dou No.65
IGDB-295	Improved	*G. max* (L.) Merr.	Fen Dou No.63
IGDB-296	Improved	*G. max* (L.) Merr.	Dong Nong No.52
IGDB-297	Improved	*G. max* (L.) Merr.	Dong Nong No.51
IGDB-298	Improved	*G. max* (L.) Merr.	Dong Nong No.26
IGDB-299	Improved	*G. max* (L.) Merr.	Chang Nong No.16
IGDB-300	Improved	*G. max* (L.) Merr.	Chang Nong No.15
IGDB-301	Improved	*G. max* (L.) Merr.	Chang Nong No.13
IGDB-302	Improved	*G. max* (L.) Merr.	Cang Dou-11
